# Fostering implementation of health services research findings into practice: a consolidated framework for advancing implementation science

**DOI:** 10.1186/1748-5908-4-50

**Published:** 2009-08-07

**Authors:** Laura J Damschroder, David C Aron, Rosalind E Keith, Susan R Kirsh, Jeffery A Alexander, Julie C Lowery

**Affiliations:** 1 HSR&D Center for Clinical Management Research, VA Ann Arbor Healthcare System (11H), 2215 Fuller Rd, Ann Arbor, MI 48105, USA; 2 VA HSR&D Center for Quality Improvement Research (14W), Louis Stokes Cleveland DVAMC, 10701 East Blvd, Cleveland, OH 44106, USA; 3 Health Management and Policy, School of Public Health, University of Michigan,109 S. Observatory (M3507 SPH II), Ann Arbor, Michigan 48109-2029, USA

## Abstract

**Background:**

Many interventions found to be effective in health services research studies fail to translate into meaningful patient care outcomes across multiple contexts. Health services researchers recognize the need to evaluate not only summative outcomes but also formative outcomes to assess the extent to which implementation is effective in a specific setting, prolongs sustainability, and promotes dissemination into other settings. Many implementation theories have been published to help promote effective implementation. However, they overlap considerably in the constructs included in individual theories, and a comparison of theories reveals that each is missing important constructs included in other theories. In addition, terminology and definitions are not consistent across theories. We describe the Consolidated Framework For Implementation Research (CFIR) that offers an overarching typology to promote implementation theory development and verification about what works where and why across multiple contexts.

**Methods:**

We used a snowball sampling approach to identify published theories that were evaluated to identify constructs based on strength of conceptual or empirical support for influence on implementation, consistency in definitions, alignment with our own findings, and potential for measurement. We combined constructs across published theories that had different labels but were redundant or overlapping in definition, and we parsed apart constructs that conflated underlying concepts.

**Results:**

The CFIR is composed of five major domains: intervention characteristics, outer setting, inner setting, characteristics of the individuals involved, and the process of implementation. Eight constructs were identified related to the intervention (*e.g*., evidence strength and quality), four constructs were identified related to outer setting (*e.g*., patient needs and resources), 12 constructs were identified related to inner setting (*e.g*., culture, leadership engagement), five constructs were identified related to individual characteristics, and eight constructs were identified related to process (*e.g*., plan, evaluate, and reflect). We present explicit definitions for each construct.

**Conclusion:**

The CFIR provides a pragmatic structure for approaching complex, interacting, multi-level, and transient states of constructs in the real world by embracing, consolidating, and unifying key constructs from published implementation theories. It can be used to guide formative evaluations and build the implementation knowledge base across multiple studies and settings.

## 

'To see far is one thing, going there is another' Constantin Brancusi, 1876–1957

## Background

Many interventions found to be effective in health services research studies fail to translate into meaningful patient care outcomes across multiple contexts. In fact, some estimates indicate that two-thirds of organizations' efforts to implement change fail [1]. Barriers to implementation may arise at multiple levels of healthcare delivery: the patient level, the provider team or group level, the organizational level, or the market/policy level [2]. Researchers must recognize the need to evaluate not only summative endpoint health outcomes, but also to perform formative evaluations to assess the extent to which implementation is effective in a specific context to optimize intervention benefits, prolong sustainability of the intervention in that context, and promotes dissemination of findings into other contexts [3]. Health services researchers are increasingly recognizing the critical role of implementation science [4]. For example, the United States Veterans Health Administration (VHA) established the Quality Enhancement Research Initiative (QUERI) in 1998 to 'systematically [implement]...clinical research findings and evidence-based recommendations into routine clinical practice' [5,6] and The National Institute for Health Research Service Delivery and Organisation Program was established to '...promote the uptake and application of...evidence in policy and practice' in the United Kingdom.

Many implementation theories to promote effective implementation have been described in the literature but have differing terminologies and definitions. A comparison of theories reveals considerable overlap, yet each is missing one or more key constructs included in other theories. A comprehensive framework that consolidates constructs found in the broad array of published theories can facilitate the identification and understanding of the myriad potentially relevant constructs and how they may apply in a particular context. Our goal, therefore, is to establish the Consolidated Framework for Implementation Research (CFIR) that comprises common constructs from published implementation theories. We describe a theoretical framework that embraces, not replaces, the significant and meaningful contribution of existing research related to implementation science.

The CFIR is 'meta-theoretical'–it includes constructs from a synthesis of existing theories, without depicting interrelationships, specific ecological levels, or specific hypotheses. Many existing theories propose 'what works' but more research is needed into what works where and why [7]. The CFIR offers an overarching typology–a list of constructs to promote theory development and verification about what works where and why across multiple contexts. Researchers can select constructs from the CFIR that are most relevant for their particular study setting and use these to guide diagnostic assessments of implementation context, evaluate implementation progress, and help explain findings in research studies or quality improvement initiatives. The CFIR will help advance implementation science by providing consistent taxonomy, terminology, and definitions on which a knowledge base of findings across multiple contexts can be built.

## Methods

Developing a comprehensive framework is more challenging than simply combining constructs from existing theories. We have carefully reviewed terminology and constructs associated with published theories for this first draft of the CFIR. In the process of standardizing terminology, we have combined certain constructs across theories while separating and delineating others to develop definitions that can be readily operationalized in implementation research studies.

We sought theories (we use the term theory to collectively refer to published models, theories, and frameworks) that facilitate translation of research findings into practice, primarily within the healthcare sector. Greenhalgh *et al*.'s synthesis of nearly 500 published sources across 13 fields of research culminated in their 'Conceptual model for considering the determinants of diffusion, dissemination, and implementation of innovations in health service delivery and organization' [8] and this was our starting point for the CFIR. We used a snowball sampling approach to identify new articles through colleagues engaged in implementation research and theories that cited Greenhalgh *et al*.'s synthesis, or that have been used in multiple published studies in health services research (*e.g*., the Promoting Action on Research Implementation in Health Services (PARiHS) framework [9]). We included theories related to dissemination, innovation, organizational change, implementation, knowledge translation, and research uptake that have been published in peer reviewed journals (one exception to this is Fixsen *et al*.'s review published by the National Implementation Research Network because of its scope and depth [10]). We did not include practice models such as the Chronic Care Model (CCM) because this describes a care delivery system, not a model for implementation [11]. The CFIR can be used to guide implementation of interventions that target specific components of the CCM.

With few exceptions, we limited our review to theories that were developed based on a synthesis of the literature or as part of a large study. Our search for implementation theories was not exhaustive but we did reach 'theme saturation': the last seven models we reviewed did not yield new constructs, though some descriptions were altered slightly with additional insights. We expect the CFIR to continue to evolve as researchers use the CFIR and contribute to the knowledge base.

The CFIR is a framework, which reflects a '...professional consensus within a particular scientific community. It stands for the entire constellation of beliefs, values, and techniques shared by members of that community... [and] need not specify the direction of relationships or identify critical hypotheses' [12].

It is important to note the last clause: The CFIR specifies a list of constructs within general domains that are believed to influence (positively or negatively, as specified) implementation, but does not specify the interactions between those constructs. The CFIR does provide a pragmatic organization of constructs upon which theories hypothesizing specific mechanisms of change and interactions can be developed and tested empirically.

Table 1 lists the theories we reviewed for inclusion into the CFIR. Greenhalgh *et al*.'s synthesis [8] was developed based on an exhaustive synthesis of a wide range of literatures including foundational work by Van de Ven, Rogers, Damanpour, and others. This body of work is an important foundation for the CFIR, though not explicitly listed in Table 1. Constructs were selected for inclusion based on strength of conceptual or evidential support in the literature for influencing implementation, high consistency in definitions, alignment with our own experience, and potential for operationalization as measures.

**Table 1:** Citation List of Models Analyzed for the CFIR

**1**	**Conceptual Model for Considering the Determinants of Diffusion, Dissemination, and Implementation of Innovations in Health Service Delivery and Organization** Greenhalgh T, Robert G, Macfarlane F, Bate P, Kyriakidou O: **Diffusion of innovations in service organizations: systematic review and recommendations**. *Milbank Q *2004, **82:**581–629.
**2**	**Conceptual Model for Implementation Effectiveness** Klein KJ, Sorra JS: The Challenge of Innovation Implementation. The Academy of Management Review 1996, 21:1055–1080. Klein KJ, Conn AB, Sorra JS: Implementing computerized technology: An organizational analysis. J Appl Psychol 2001, 86:811–824.

**3**	**Dimensions of Strategic Change** Pettigrew A, Whipp R: **Managing change and corporate performance**. In *European Industrial Restructuring in the 1990s*. Edited by Cool K, Neven DJ, Walter I. Washington Square, NY: New York University Press; 1992: 227–265

**4**	**Theory-based Taxonomy for Implementation** Leeman J, Baernholdt M, Sandelowski M: **Developing a theory-based taxonomy of Methods for implementing change in practice**. *J Adv Nurs *2007, 58:191–200.

**5**	**PARiHS Framework: Promoting Action on Research Implementation in Health Services** Kitson A: **From research to practice: one organisational model for promoting research based practice**. *Edtna Erca J *1997, **23:**39–45. Rycroft-Malone J, Harvey G, Kitson A, McCormack B, Seers K, Titchen A: **Getting evidence into practice: ingredients for change**. *Nurs Stand *2002, **16:**38–43.

**6**	**Ottawa Model of Research Use** Graham ID, Logan J: **Innovations in knowledge transfer and continuity of care**. *Can J Nurs Res *2004, **36:**89–103.

**7**	**Conceptual Framework for Transferring Research to Practice** Simpson DD: **A conceptual framework for transferring research to practice**. *J Subst Abuse Treat *2002, **22:**171–182. Simpson DD, Dansereau DF: **Assessing Organizational Functioning as a Step Toward Innovation**. *NIDA Science and Practice Perspectives *2007, **3:**20–28.

**8**	**Diagnositic/Needs Assessment** Kochevar LK, Yano EM: **Understanding health care organization needs and context. Beyond performance gaps**. *J Gen Intern Med *2006, **21 Suppl 2:**S25–29.

**9**	**Stetler Model of Research Utilization** Stetler CB: **Updating the Stetler Model of research utilization to facilitate evidence-based practice**. *Nurs Outlook *2001, **49:**272–279.

**10**	**Technology Implementation Process Model** Edmondson AC, Bohmer RM, Pisana GP: **Disrupted routines: Team learning and new technology implementation in hospitals**. *Adm Sci Q *2001, **46:**685–716.

**11**	**Replicating Effective Programs Framework** Kilbourne AM, Neumann MS, Pincus HA, Bauer MS, Stall R: **Implementing evidence-based interventions in health care: Application of the replicating effective programs framework**. *Implement Sci *2007, **2:**42.

**12**	**Organizational Transformation Model** VanDeusen Lukas CV, Holmes SK, Cohen AB, Restuccia J, Cramer IE, Shwartz M, Charns MP: **Transformational change in health care systems: An organizational model**. *Health Care Manage Rev *2007, **32:**309–320.

**13**	**Implementation of Change: A Model** Grol RP, Bosch MC, Hulscher ME, Eccles MP, Wensing M: **Planning and studying improvement in patient care: the use of theoretical perspectives**. *Milbank Q *2007, **85:**93–138. Grol R, Wensing M, Eccles M: *Improving Patient Care: The Implementation of Change in Clinical Practice*. Edinburgh, Scotland: Elsevier; 2005.

**14**	**Framework of Dissemination in Health Services Intervention Research** Mendel P, Meredith LS, Schoenbaum M, Sherbourne CD, Wells KB: **Interventions in organizational and community context: a framework for building evidence on dissemination and implementation in health services research**. *Adm Policy Ment Health *2008, **35:**21–37.

**15**	**Conceptual Framework for Implementation of Defined Practices and Programs** Fixsen DL, Naoom, S. F., Blase, K. A., Friedman, R. M. and Wallace, F.: **Implementation Research: A Synthesis of the Literature**. (The National Implementation Research Network ed.: University of South Florida, Louis de la Parte Florida Mental Health Institute; 2005.

**16**	**Will it Work Here? A Decision-maker's Guide Adopting Innovations** Brach C, Lenfestey N, Roussel A, Amoozegar J, Sorensen A: *Will It Work Here? A Decisionmaker's Guide to Adopting Innovations*. Agency for Healthcare Research and Quality (AHRQ); 2008.

**17**	**Availability, Responsiveness and Continuity: An Organizational and Community Intervention Model** Glisson C, Schoenwald SK: **The ARC organizational and community intervention strategy for implementing evidence-based children's mental health treatments**. *Ment Health Serv Res *2005, **7:**243–259. Glisson C, Landsverk J, Schoenwald S, Kelleher K, Hoagwood KE, Mayberg S, Green P: **Assessing the Organizational Social Context (OSC) of Mental Health Services: Implications for Research and Practice**. *Adm Policy Ment Health *2008, **35:**98–113.

**18**	**A Practical, Robust Implementation and Sustainability Model (PRISM)** Feldstein AC, Glasgow RE: **A practical, robust implementation and sustainability model (PRISM) for integrating research findings into practice**. *Jt Comm J Qual Patient Saf *2008, **34:**228–243.

**19**	**Multi-level Conceptual Framework of Organizational Innovation Adoption** Frambach RT, Schillewaert N: **Organizational innovation adoption: a multi-level framework of determinants and opportunities for future research**. *Journal of Business Research *2001, **55:**163–176.

### Foundational definitions

Implementation, context, and setting are concepts that are widely used and yet have inconsistent definitions and usage in the literature; thus, we present working definitions for each. Implementation is the constellation of processes intended to get an intervention into use within an organization [13]; it is the means by which an intervention is assimilated into an organization. Implementation is the critical gateway between an organizational decision to adopt an intervention and the routine use of that intervention; the transition period during which targeted stakeholders become increasingly skillful, consistent, and committed in their use of an intervention [14].

Implementation, by its very nature, is a social process that is intertwined with the context in which it takes place [15]. Context consists of a constellation of active interacting variables and is not just a backdrop for implementation [16]. For implementation research, 'context' is the set of circumstances or unique factors that surround a particular implementation effort. Examples of contextual factors include a provider's perception of the evidence supporting the use of a clinical reminder for obesity, local and national policies about how to integrate that reminder into a local electronic medical record, and characteristics of the individuals involved in the implementation effort. The theories underpinning the intervention and implementation [17] also contribute to context. In this paper, we use the term context to connote this broad scope of circumstances and characteristics. The 'setting' includes the environmental characteristics in which implementation occurs. Most implementation theories in the literature use the term context both to refer to broad context, as described above, and also the specific setting.

## Results

### Overview of the CFIR

The CFIR comprises five major domains (the intervention, inner and outer setting, the individuals involved, and the process by which implementation is accomplished). These domains interact in rich and complex ways to influence implementation effectiveness. More than 20 years ago, Pettigrew and Whipp emphasized the essential interactive dimensions of content of intervention, context (inner and outer settings), and process of implementation [18]. This basic structure is also echoed by the PARiHS framework that describes three key domains of evidence, context, and facilitation [9]. Fixsen, *et al*. emphasize the multi-level influences on implementation, from external influencers to organizational and core implementation process components, which include the central role of the individuals who coach and train prospective practitioners and the practitioners themselves [10].

The first major domain of the CFIR is related to characteristics of the intervention being implemented into a particular organization. Without adaptation, interventions usually come to a setting as a poor fit, resisted by individuals who will be affected by the intervention, and requiring an active process to engage individuals in order to accomplish implementation. The intervention is often complex and multi-faceted, with many interacting components. Interventions can be conceptualized as having 'core components' (the essential and indispensible elements of the intervention) and an 'adaptable periphery' (adaptable elements, structures, and systems related to the intervention and organization into which it is being implemented) [8,10]. For example, a clinical reminder to screen for obesity has an alert that pops up on the computer screen at the appropriate time for the appropriate patient. This feature is part of the core of the intervention. Just as importantly, the intervention's adaptable periphery allows it to be modified to the setting without undermining the integrity of that intervention. For example, depending on the work processes at individual clinics, the clinical reminder could pop up during the patient assessment by a nurse case manager or when the primary care provider evaluates the patient. Components of the periphery can be modified to a particular setting and vice versa in a co-evolving/co-adaptive way [19,20].

The next two domains in the CFIR are inner and outer setting. Changes in the outer setting can influence implementation, often mediated through changes in the inner setting [21]. Generally, the outer setting includes the economic, political, and social context within which an organization resides, and the inner setting includes features of structural, political, and cultural contexts through which the implementation process will proceed [22]. However, the line between inner and outer setting is not always clear and the interface is dynamic and sometimes precarious. The specific factors considered 'in' or 'out' will depend on the context of the implementation effort. For example, outlying clinics may be part of the outer setting in one study, but part of the inner setting in another study. The inner setting may be composed of tightly or loosely coupled entities (*e.g*., a loosely affiliated medical center and outlying contracted clinics or tightly integrated service lines within a health system); tangible and intangible manifestation of structural characteristics, networks and communications, culture, climate, and readiness all interrelate and influence implementation.

The fourth major domain of the CFIR is the individuals involved with the intervention and/or implementation process. Individuals have agency; they make choices and can wield power and influence on others with predictable or unpredictable consequences for implementation. Individuals are carriers of cultural, organizational, professional, and individual mindsets, norms, interests, and affiliations. Greenhalgh *et al*. describe the significant role of individuals [8]:

'People are not passive recipients of innovations. Rather...they seek innovations, experiment with them, evaluate them, find (or fail to find) meaning in them, develop feelings (positive or negative) about them, challenge them, worry about them, complain about them, 'work around' them, gain experience with them, modify them to fit particular tasks, and try to improve or redesign them–often through dialogue with other users.'

Many theories of individual change have been published [23], but little research has been done to gain understanding of the dynamic interplay between individuals and the organization within which they work, and how that interplay influences individual or organizational behavior change. One recent synthesis of 76 studies using social cognitive theories of behavior change found that the Theory of Planned Behavior (TPB) model was the most often used model to explain intention and predict clinical behavior of health professionals. The TPB, overall, succeeded in explaining 31% of variance in behavior [24]. The authors suggest that 'special care' is needed to better define (and understand) the context of behavioral performance. Frambach and Schillewaert's multi-level framework is unique in explicitly acknowledging the multi-level nature of change by integrating individual behavior change within the context of organizational change [25]. Individuals in the inner setting include targeted users and other affected individuals.

The fifth major domain is the implementation process. Successful implementation usually requires an active change process aimed to achieve individual and organizational level use of the intervention as designed. Individuals may actively promote the implementation process and may come from the inner or outer setting (*e.g*., local champions, external change agents). The implementation process may be an interrelated series of sub-processes that do not necessarily occur sequentially. There are often related processes progressing simultaneously at multiple levels within the organization [22]. These sub-processes may be formally planned or spontaneous; conscious or subconscious; linear or nonlinear, but ideally are all aimed in the same general direction: effective implementation.

In summary, the CFIR's overarching structure supports the exploration of essential factors that may be encountered during implementation through formative evaluations [3,26]. Additional File 1 contains a figure that visually depicts the five interrelated major domains. Using the five major domains as an initial organizing structure (*i.e*., intervention, outer and inner setting, individuals involved, and process), we mapped the broad array of constructs described in Greenhalgh, *et al*.'s conceptual model and the 18 additional theories listed in Table 1 to constructs in the CFIR.

### Detailed description of CFIR constructs

Some constructs appear in many of the theories included in the CFIR (*e.g*., available resources appears in 10 of the 19 theories we reviewed), while others are more sparsely supported (*e.g*., cost of the intervention only appears in five of the 19 theories). Additional File 2 provides a table that lists each published theory and the constructs included in each theory. Additional File 3 provides a quick reference table that lists each construct, along with a short definition. Additional File 4 provides detailed rationale for each construct.

Evaluation of most of the constructs relies on individual perceptions. For example, it is one thing for an outside expert panel to rate an intervention as having 'gold standard' level of evidence supporting its use. Stakeholders in the receiving organization may have an entirely different perception of that same evidence. It is the latter perceptions, socially constructed in the local setting, which will affect implementation effectiveness. It is thus important to design formative evaluations that carefully consider how to elicit, construct, and interpret findings to reflect the perceptions of the individuals and their organization, not just the perceptions or judgments of outside researchers or experts.

### Intervention characteristics

#### Intervention source

Perception of key stakeholders about whether the intervention is externally or internally developed [8]. An intervention may be internally developed as a good idea, solution to a problem, or other grass-roots effort, or may be developed by an external entity (*e.g*., vendor or research group) [8]. The legitimacy of the source may also influence implementation.

#### Evidence strength and quality

Stakeholders' perceptions of the quality and validity of evidence supporting the belief that the intervention will have desired outcomes. Sources of evidence may include published literature, guidelines, anecdotal stories from colleagues, information from a competitor, patient experiences, results from a local pilot, and other sources [9,27].

#### Relative advantage

Stakeholders' perception of the advantage of implementing the intervention versus an alternative solution [28].

#### Adaptability

The degree to which an intervention can be adapted, tailored, refined, or reinvented to meet local needs. Adaptability relies on a definition of the 'core components' (the essential and indispensible elements of the intervention itself) versus the 'adaptable periphery' (adaptable elements, structures, and systems related to the intervention and organization into which it is being implemented) of the intervention [8,10], as described in the Overview section. A component analysis can be performed to identify the core versus adaptable periphery components [29], but often the distinction is one that can only be discerned through trial and error over time as the intervention is disseminated more widely and adapted for a variety of contexts [26]. The tension between the need to achieve full and consistent implementation across multiple contexts while providing the flexibility for local sites to adapt the intervention as needed is real and must be balanced, which is no small challenge [30].

#### Trialability

The ability to test the intervention on a small scale in the organization [8], and to be able to reverse course (undo implementation) if warranted [31]. The ability to trial is a key feature of the plan-do-study-act quality improvement cycle that allows users to find ways to increase coordination to manage interdependence [32]. Piloting allows individuals and groups to build experience and expertise, and time to reflect upon and test the intervention [33], and usability testing (with staff and patients) promotes successful adaptation of the intervention [31].

#### Complexity

Perceived difficulty of implementation, reflected by duration, scope, radicalness, disruptiveness, centrality, and intricacy and number of steps required to implement [8,23]. Radical interventions require significant reorientation and non-routine processes to produce fundamental changes in the organization's activities and reflects a clear departure from existing practices [8]. One way to determine complexity is by assessing 'length' (the number of sequential sub-processes or steps for using or implementing an intervention) and 'breadth' (number of choices presented at decision points) [34]. Complexity is also increased with higher numbers of potential target organizational units (teams, clinics, departments) or types of people (providers, patients, managers) targeted by the intervention [34], and the degree to which the intervention will alter central work processes [23].

#### Design quality and packaging

Perceived excellence in how the intervention is bundled, presented, and assembled [35].

#### Cost

Costs of the intervention and costs associated with implementing that intervention, including investment, supply, and opportunity costs. It is important to differentiate this construct from available resources (part of inner setting, below). In many contexts, costs are difficult to capture and available resources may have a more direct influence on implementation.

### Outer setting

#### Patient needs and resources

The extent to which patient needs, as well as barriers and facilitators to meet those needs, are accurately known and prioritized by the organization. Clearly, improving the health and well-being of patients is the mission of all healthcare entities, and many calls have gone out for organizations to be more patient centered [21]. Patient-centered organizations are more likely to implement change effectively [36]. Many theories of research uptake or implementation acknowledge the importance of accounting for patient characteristics [31,33,37], and consideration of patients needs and resources must be integral to any implementation that seeks to improve patient outcomes [21]. The Practical, Robust Implementation and Sustainability Model PRISM delineates six elements that can help guide evaluation of the extent to which patients are at the center of organizational processes and decisions: patient choices are provided, patient barriers are addressed, transition between program elements is seamless, complexity and costs are minimized, and patients have high satisfaction with service and degree of access and receive feedback [31].

#### Cosmopolitanism

The degree to which an organization is networked with other external organizations. Organizations that support and promote external boundary-spanning roles of their staff are more likely to implement new practices quickly [8]. The collective networks of relationships of individuals in an organization represent the social capital of the organization [38]. Social capital is one term used to describe the quality and the extent of those relationships and includes dimensions of shared vision and information sharing. One component of social capital is external bridging between people or groups outside the organization [8].

#### Peer pressure

Mimetic or competitive pressure to implement an intervention, typically because most or other key peer or competing organizations have already implemented or in pursuit of a competitive edge. 'Peers' can refer to any outside entity with which the organization feels some degree of affinity or competition at some level within their organization (*e.g*., competitors in the market, other hospitals in a network). The pressure to implement can be particularly strong for late-adopting organizations [39].

#### External policies and incentives

Broad constructs that encompass external strategies to spread interventions, including policy and regulations (governmental or other central entity), external mandates, recommendations and guidelines, pay-for-performance, collaboratives, and public or benchmark reporting [26].

### Inner setting

Contributing to the complexity inherent in describing the many constructs related to the inner setting, are challenges inherent in conceptualizing the myriad levels in which these constructs influence and interact. Little systematic research has been done to understand how constructs apply to different levels within an organization, whether constructs apply equally to all levels, and which constructs are most important at which level.

#### Structural characteristics

The social architecture, age, maturity, and size of an organization. Social architecture describes how large numbers of people are clustered into smaller groups and differentiated, and how the independent actions of these differentiated groups are coordinated to produce a holistic product or service [40]. Structural characteristics are, by-and-large, quantitative measures and, in most cases, measurement instruments and approaches have been developed for them. Damenpour conducted a meta-analysis of many structural determinants based on 23 studies conducted outside the healthcare sector [41]. Functional differentiation is the internal division of labor where coalitions of professionals are formed into differentiated units. The number of units or departments represents diversity of knowledge in an organization. The more stable teams are (members are able to remain with the team for an adequate period of time; low turnover), the more likely implementation will be successful [42]. Administrative intensity (the ratio of managers to total employees) is positively associated with innovation [41]. Centralization (the concentration of decision-making autonomy) has been shown to be negatively associated with innovation [41], but has also been found to be positive or negatively associated, depending on the stage of intervention (initiative stage versus implementation stage) [43]. Size, age, maturity, and degree of specialization (the uniqueness of the niche or market for the organization's products or services) also influence implementation [8].

#### Networks and communications

The nature and quality of webs of social networks and the nature and quality of formal and informal communications within an organization. Research on organizational change has moved beyond reductionist measures of organizational structure, and increasingly embraces the complex role that networks and communications have on implementation of change interventions [44]. Connections between individuals, units, services, and hierarchies may be strong or weak, formal or informal, tangible or intangible. Social capital describes the quality and the extent of relationships and includes dimensions of shared vision and information sharing. One component of social capital is the internal bonding of individuals within the same organization [8]. Complexity theory posits that relationships between individuals may be more important than individual attributes [45], and building these relationships can positively influence implementation [46]. These relationships may manifest to build a sense of 'teamness' or 'community' that may contribute to implementation effectiveness [42].

Regardless of how an organization is structurally organized, the importance of communication across the organization is clear. Communication failures are involved with the majority of sentinel events in US hospitals [47]. High quality of formal communications contributes to effective implementation [48]. Making staff feel welcome (good assimilation), peer collaboration and open feedback and review among peers and across hierarchical levels, clear communication of mission and goals, and cohesion between staff and informal communication quality, all contribute to effective implementation [48].

#### Culture

Norms, values, and basic assumptions of a given organization [49]. Most change efforts are targeted at visible, mostly objective, aspects of an organization that include work tasks, structures, and behaviors. One explanation for why so many of these initiatives fail centers on the failure to change less tangible organizational assumptions, thinking, or culture [50].

Some researchers have a relatively narrow definition of culture, while other researchers incorporate nearly every construct related to inner setting. In the next section we highlight the concept of 'climate.' As with 'culture,' climate suffers from inconsistent definition. Culture and climate can, at times, be interchangeable across studies, depending on the definition used [51]. A recent review found 54 different definitions for organizational climate [49] and, likewise, many definitions exist for culture [51]. Culture is often viewed as relatively stable, socially constructed, and subconscious [51]. The CFIR embraces this latter view and differentiates climate as the localized and more tangible manifestation of the largely intangible, overarching culture [49]. Climate is a phenomenon that can vary across teams or units, and is typically less stable over time compared to culture.

#### Implementation climate

The absorptive capacity for change, shared receptivity of involved individuals to an intervention [8], and the extent to which use of that intervention will be 'rewarded, supported, and expected within their organization' [14]. Climate can be assessed through tangible and relatively accessible means such as policies, procedures, and reward systems [49]. Six sub-constructs contribute to a positive implementation climate for an intervention: tension for change, compatibility, relative priority, organizational incentives and rewards, goals and feedback, and learning climate.

1. Tension for change: The degree to which stakeholders perceive the current situation as intolerable or needing change [8,48].

2. Compatibility: The degree of tangible fit between meaning and values attached to the intervention by involved individuals, how those align with individuals' own norms, values, and perceived risks and needs, and how the intervention fits with existing workflows and systems [8,14]. The more individuals perceive alignment between the meaning they attach to the intervention and meaning communicated by upper management, the more effective implementation is likely to be. For example, providers may perceive an intervention as a threat to their autonomy, while leadership is motivated by the promise of better patient outcomes.

3. Relative priority: Individuals' shared perception of the importance of the implementation within the organization [14,31,35].

4. Organizational incentives and rewards: Extrinsic incentives such as goal-sharing awards, performance reviews, promotions, and raises in salary, as well as less tangible incentives such as increased stature or respect [35,52].

5. Goals and feedback: The degree to which goals are clearly communicated, acted upon, and fed back to staff and alignment of that feedback with goals [34,48,53]. The Chronic Care Model emphasizes the importance of relying on multiple methods of evaluation and feedback including clinical, performance, and economic evaluations and experience [11].

6. Learning climate: A climate in which: leaders express their own fallibility and need for team members' assistance and input; team members feel that they are essential, valued, and knowledgeable partners in the change process; individuals feel psychologically safe to try new methods; and there is sufficient time and space for reflective thinking and evaluation (in general, not just in a single implementation) [14,35,54]. These interrelated practices and beliefs support and enable employee and organizational skill development, learning, and growth to maximize an organization's absorptive capacity for new knowledge and methods [8]. Quantitative measurement instruments are available for measuring an organization's 'learning' capability [55].

Readiness for implementation: Tangible and immediate indicators of organizational commitment to its decision to implement an intervention, consisting of three sub-constructs (leadership engagement, available resources, and access to information and knowledge). Implementation readiness is differentiated from implementation climate in the literature by its inclusion of specific tangible and immediate indicators of organizational commitment to its decision to implement an intervention. Additional File 4 provides more discussion and rationale for the constellation and grouping of sub-constructs for implementation climate and readiness for implementation.

1. Leadership engagement: Commitment, involvement, and accountability of leaders and managers [35,53] with the implementation. The term 'leadership' can refer to leaders at any level of the organization, including executive leaders, middle management, front-line supervisors, and team leaders, who have a direct or indirect influence on the implementation. One important dimension of organizational commitment is managerial patience (taking a long-term view rather than short-term) to allow time for the often inevitable reduction in productivity until the intervention takes hold [35].

2. Available resources: The level of resources dedicated for implementation and ongoing operations including money, training, education, physical space, and time [8,28,42,48,56,57].

3. Access to information and knowledge: Ease of access to digestible information and knowledge about the intervention and how to incorporate it into work tasks [8]. Information and knowledge includes all sources such as experts, other experienced staff, training, documentation, and computerized information systems.

### Characteristics of individuals

Little research has been done to gain understanding of the dynamic interplay between individuals and the organization within which they work and how that interplay influences individual or organizational behavior change. Organizations are, fundamentally, composed of individuals. However, the problem of the level of analysis is particularly clear when describing individual characteristics. Though the characteristics described here are necessarily measured at the individual level, these measures may be most appropriately aggregated to team or unit or service levels in analyses. The level at which to perform analysis is determined by the study context. For example, VanDeusen Lukas, *et al*. measured knowledge and skills at an individual level, but then aggregated this measure to the team level in their study of factors influencing implementation of an intervention in ambulatory care clinics [58]. Organizational change starts with individual behavior change. Individual knowledge and beliefs toward changing behavior and the level of self-efficacy to make the change have been widely studied and are the two most common individual measures in theories of individual change [23]. The CFIR includes these two constructs along with individual identification with the organization and other personal attributes.

#### Knowledge and beliefs about the intervention

Individuals' attitudes toward and value placed on the intervention, as well as familiarity with facts, truths, and principles related to the intervention. Skill in using the intervention is a primarily cognitive function that relies on adequate how-to knowledge and knowledge of underlying principles or rationale for adopting the intervention [59]. Enthusiastic use of an intervention can be reflected by a positive affective response to the intervention. Often, subjective opinions obtained from peers based on personal experiences are more accessible and convincing, and these opinions help to generate enthusiasm [59]. Of course, the converse is true as well, often creating a negative source of active or passive resistance [60]. The degree to which new behaviors are positively or negatively valued heightens intention to change, which is a precursor to actual change [61].

#### Self-efficacy

Individual belief in their own capabilities to execute courses of action to achieve implementation goals [62]. Self-efficacy is a significant component in most individual behavior change theories [63]. Self-efficacy is dependent on the ability to perform specific actions within a specific context. The more confident an individual feels about his or her ability to make the changes needed to achieve implementation goals, the higher their self-efficacy. Individuals with high self-efficacy are more likely to make a decision to embrace the intervention and exhibit committed use even in the face of obstacles.

#### Individual stage of change

Characterization of the phase an individual is in, as he or she progresses toward skilled, enthusiastic, and sustained use of the intervention [23,35]. The specific stages used will depend on the underlying model being used in the study. Prochaska's trans-theoretical model characterizes these stages as pre-contemplation, contemplation, preparation, and action and maintenance [64]. Rogers' diffusion theory delineates five stages [59]. Grol *et al*. describe a five-stage model with ten sub-stages based on their synthesis of the literature [23].

#### Individual identification with organization

A broad construct related to how individuals perceive the organization and their relationship and degree of commitment to that organization. These attributes may affect the willingness of staff to fully engage in implementation efforts or use the intervention [65,66]. These measures have been studied very little in healthcare, but may be especially important when evaluating the influence of implementation leaders' (described under Process below) on implementation efforts. Organizational citizenship behavior characterizes how well organizational identity is taken on by individuals and whether, because they associate themselves with the organization, they are willing to put in extra effort, talk well of the organization, and take risks in their organization [67,68]. Organizational justice is an individual's perception of distributive and procedural fairness in the organization [65]. Emotional exhaustion is an ongoing state of emotional and physical depletion or burnout [69], and may negatively influence implementation by stunting the ability and energy of an individual to help or initiate change [70]. The Agency for Healthcare Research and Quality recently published a guide for determining whether a particular implementation will be successful that includes questions about individual perceptions of whether they believe the organization could be doing a better job, belief about whether work is done efficiently, and whether there are inequities as potential barriers to implementation [71]. The organizational social context measure, developed by Glisson *et al*., includes constructs related to psychological climate (perception of the psychological influence of work environment) and work attitudes (job satisfaction and organizational commitment) [72].

#### Other personal attributes

This is a broad construct to include other personal traits. Traits such as tolerance of ambiguity, intellectual ability, motivation, values, competence, capacity, innovativeness [25], tenure [25], and learning style have not received adequate attention by implementation researchers [8].

### Process

We describe four essential activities of implementation process that are common across organizational change models: planning, engaging, executing, and reflecting and evaluating. These activities may be accomplished formally or informally through, for example, grassroots change efforts. They can be accomplished in any order and are often done in a spiral, stop-and-start, or incremental approach to implementation [73]; *e.g*., using a plan-do-study-act approach to incremental testing [74]. Each activity can be revisited, expanded, refined, and re-evaluated throughout the course of implementation.

#### Planning

The degree to which a scheme or method of behavior and tasks for implementing an intervention are developed in advance and the quality of those schemes or methods. The fundamental objective of planning is to design a course of action to promote effective implementation by building local capacity for using the intervention, collectively and individually [26]. The specific steps in plans will be based on the underlying theories or models used to promote change at organization and individual levels [23]. For example, the Institute for Healthcare Improvement [74,75], Grol *et al*. [76], and Glisson and Schoenwald [77] all describe comprehensive approaches to implementation on which implementation plans can be developed. However, these theories prescribe different sets of activities because they were developed in different contexts–though commonalities exist as well. Grol *et al*. list 14 different bodies of theories for changing behaviors in social or organizational contexts [23], and Estabrooks *et al*. list 18 different models of organizational innovation [78]. Thus, the particular content of plans will vary depending on the theory or model being used to guide implementation. Implementation plans can be evaluated by the degree to which five considerations guide planning: stakeholders' needs and perspectives are considered; strategies are tailored for appropriate subgroups (*e.g*., delineated by professional, demographic, cultural, organizational attributes); appropriate style, imagery, and metaphors are identified and used for delivering information and education; appropriate communication channels are identified and used; progress toward goals and milestones is tracked using rigorous monitoring and evaluation methods [8,59]; and strategies are used to simplify execution. The latter step may include plans for dry runs (simulations or practice sessions) to allow team members to learn how to use the intervention before going live [42], running trials to allow users to test procedures, gain confidence, and build an environment of psychological safety [42], or taking an incremental approach that breaks the intervention down into manageable parts that can be implemented incrementally [41]. The plan can be formal or informal but should consider all salient contextual factors–both modifiable and non-modifiable. Workarounds can be developed for non-modifiable factors, and strategies can be designed to change factors that can be modified (*e.g*., increase stakeholders' knowledge of the intervention).

#### Engaging

Attracting and involving appropriate individuals in the implementation and use of the intervention through a combined strategy of social marketing, education, role modeling, training, and other similar activities. Engaging members of teams tasked with implementing an intervention (or to be 'first users') is an often overlooked part of implementation [79]. It is vital that early members are carefully and thoughtfully selected or allowed to rise naturally [42,79], especially 'implementation leaders' and 'champions.' If early users and leaders are homophilous (similar socioeconomic, professional, educational, and cultural backgrounds) with intended users, individuals will be more likely to adopt the intervention [8]. The influence of these leaders can be evaluated by assessing their presence or absence (*e.g*., does the implementation effort have a clear champion or not?), how they are brought on board (*e.g*., appointed, volunteered), their role in the organization (formal and/or informal roles), and their role in implementation. One means by which influence is transmitted is role modeling [80]. We have identified four types of implementation leaders. Terms and definitions of roles vary widely in the literature. The remainder of this section suggests standard definitions for each:

1. Opinion leaders: Individuals in an organization who have formal or informal influence on the attitudes and beliefs of their colleagues with respect to implementing the intervention [8,59]. There is general agreement that there are two different types of opinion leaders, experts and peers. Expert opinion leaders exert influence through their authority and status [8]. Peer opinion leaders exert influence through their representativeness and credibility [8].

2. Formally appointed internal implementation leaders: Individuals from within the organization who have been formally appointed with responsibility for implementing an intervention as coordinator, project manager, team leader, or other similar role. These leaders may or may not have explicit time dedicated to the task. Implementation is 'part of the job.'

3. Champions: 'Individuals who dedicate themselves to supporting, marketing, and 'driving through an [implementation]' [81], overcoming indifference or resistance that the intervention may provoke in an organization. A defining characteristic of champions is their willingness to risk informal status and reputation because they believe so strongly in the intervention [82]. The main distinction of champions from opinion leaders is that champions actively associate themselves with support of the intervention during implementation. There is the old adage that an intervention 'either finds a champion or dies' [83].

4. External change agents: Individuals who are affiliated with an outside entity who formally influence or facilitate intervention decisions in a desirable direction. They usually have professional training in a technical field related to organizational change science or in the technology being introduced into the organization. This role includes outside researchers who may be implementing a multi-site intervention study and other formally appointed individuals from an external entity (related or unrelated to the organization); *e.g*., a facilitator from a corporate or regional office or a hired consultant.

#### Executing

Carrying out or accomplishing the implementation according to plan. Execution of an implementation plan may be organic with no obvious or formal planning, which makes execution difficult to assess. Quality of execution may consist of the degree of fidelity of implementation to planned courses of action [29], intensity (quality and depth) of implementation [84], timeliness of task completion, and degree of engagement of key involved individuals (*e.g*., implementation leaders) in the implementation process.

#### Reflecting and evaluating

Quantitative and qualitative feedback about the progress and quality of implementation accompanied with regular personal and team debriefing about progress and experience. It is important to differentiate this processual construct from the Goals and Feedback construct under Inner Setting, described above. The focus here is specifically related to implementation efforts. Evaluation includes traditional forms of feedback, such as reports, graphs, and qualitative feedback and anecdotal stories of success [63]. Objectives should be specific, measurable, attainable, relevant, and timely (the SMART rubric) [71]. Less attention is paid in the literature to the need for, and value of, group and personal reflection. Dedicating time for reflecting or debriefing before, during, and after implementation is one way to promote shared learning and improvements along the way [42].

## Discussion

Process theories can be used to guide how implementation should be planned, organized, and scheduled, and impact theories can be used to develop hypotheses about how implementation activities will facilitate a desired change[23] The CFIR is a pragmatic meta-theoretical framework that can be used to complement these theories with its comprehensive taxonomy of specific constructs related to the intervention, inner and outer setting, individuals, and implementation process. For example, the CFIR complements a process theory published by Pronovost and colleagues from the Johns Hopkins Quality and Safety Research Group for large-scale translation of scientific evidence into practice that encompasses four major steps [80]. The second step in this process theory is to identify local barriers to implementation without specifying what those barriers may be; the CFIR provides a list of constructs to consider. The RE-AIM framework is used to guide comprehensive evaluation of interventions in terms of Reach, Effectiveness, Adoption, Implementation, and Maintenance (sustainability) [85]. The CFIR opens the 'black box' of the 'I' (implementation) component.

The constructs described in the CFIR represent a beginning foundation for understanding implementation. Implementation researchers should assess each construct for salience, carefully adapt and operationalize definitions for their study (paying special attention to sometimes indistinct boundaries between constructs), discern the level(s) at which each should be evaluated and defined (*e.g*., individuals, teams, units, clinics, medical centers, regions), decide how to measure and assess, and be aware of the time points at which measurement and evaluation occurs while acknowledging the transient nature of the state of each of these contextual factors. Each decision and rationale should be documented along with findings related to each construct.

An example of the importance of discerning the different levels at which constructs apply is a study conducted by Van Deusen Lukas and colleagues who found that 'clinic team knowledge and skills' was associated with effective implementation [58]. They assessed team knowledge and skills by surveying individual staff members and then aggregating to the team level as one unit of analysis in predictive models. Their final model found significant contextual factors at the system (external policy and incentives), facility (management support), and team (knowledge and skills) levels. As findings accumulate into knowledge across study contexts, implementation researchers will understand more about what works where and why and be better able to predict implementation effectiveness across disparate contexts.

The CFIR can be used to guide formative evaluations. Mendel *et al*.'s framework of dissemination describes three phases of evaluation (capacity/needs assessment, implementation/process evaluation, and outcome/impact evaluation) in an intervention study or program evaluation [26] (similar phases are also described by Stetler *et al*. [3] as diagnostic analysis, implementation- and progress-focused evaluations, and interpretive evaluation). Prior to implementation, capacity and needs assessments are done to identify potential barriers and facilitators to implement from the perspective of the individuals and organizations involved in the implementation. The CFIR provides a list of explicitly defined constructs for which data can be collected. It is important, however, that the CFIR not be applied wholesale to every problem. The long list of constructs, each with their own level of 'maturity' in definition and operability, can quickly mire evaluations. Rather, each construct should be evaluated strategically, in the context of the study or evaluation, to determine those that will be most fruitful to study [17] or that are necessary to properly adapt the intervention to the setting. For example, in a current implementation study, we are assessing the benefits of an intervention designed to improve blood pressure management for patients with diabetes. Before implementation, we chose salient constructs to guide our capacity/needs assessment which revealed differences between sites. This information was used to guide adaptation of the intervention and to develop implementation plans for each site. A simple example of adaptation is the difference in protocols across study sites for obtaining blood pressure cuffs for patients.

During implementation, it is important to monitor progress for unanticipated influences (barriers or facilitators) and progress toward implementation goals. In our blood pressure management study, baseline findings from our pre-implementation assessments led us to closely monitor whether the intervention pharmacists had ongoing and timely access to the information they needed for their patient encounters (Access to Information and Knowledge). We discovered, during the intervention, that slow-running software at one site was interfering with the clinical pharmacist's ability to communicate efficiently with patients during encounters and we were able to facilitate a timely solution. Pre-implementation assessments have allowed us to target collection of real-time data as implementation progresses to track key implementation processes, so that we can address problems before they threaten the intervention's viability. Findings are mapped to specific CFIR constructs (e.g., access to information and knowledge)

The third type of evaluation described in Mendel et al.'s model is outcome and impact evaluation. Post-implementation, the CFIR can be used to guide exploration into the question of what factors influenced implementation and how implementation influenced performance of the intervention. For example, the CFIR can be used to assess changes in the inner setting (e.g., leadership engagement) as a result of implementation the co-evolution that often occurs through effective implementation [20]. At all three evaluation stages, the CFIR provides a framework by which to understand the dynamic, multi-level, transient nature of implementation in specific contexts and to organize and communicate findings across contexts.

At a macro level, the CFIR can be used to organize and promote synthesis of research findings, studies, and settings [26] using clear and consistent language and terminology, which will further stimulate theory development. The Consolidated Standards of Reporting Trials (CONSORT) Trial Bank Project was developed to do this for clinical trials by capturing study design, execution details, and results from randomized clinical trials in a form that promotes synthesis of results from multiple studies [86]. The recently published Standards for QUality Improvement Reporting Excellence (SQUIRE) guidelines are designed to promote knowledge-building for implementation and quality improvement studies by standardizing how findings from these studies are reported. The SQUIRE guidelines take into account two essential considerations missing from the CONSORT guidelines but essential for implementation studies: 'reflexivity' and setting [15]. The guidelines suggest that authors specify, '...how elements of the local care environment considered most likely to influence change/improvement in the involved site or sites were identified and characterized' [15]. Constructs included in the CFIR can be used to explicate those elements more consistently across studies.

The ultimate judgment of the CFIR's utility and validity can be discerned by coalescing answers to three questions over time [12]:

1. Is terminology and language coherent?

2. Does the CFIR promote comparison of results across contexts and studies over time?

3. Does the CFIR stimulate new theoretical developments?

If answers to all three questions are yes, then we are on the right path.

## Conclusion

The CFIR provides a pragmatic structure for identifying potential influences on implementation and organizing findings across studies. It embraces, consolidates, standardizes, and unifies constructs shown to be associated with implementation from other published implementation theories. The CFIR can be used to help guide formative evaluations of interventions in context and offers an organizational framework for synthesizing and building knowledge about what works where, across multiple settings. We propose the CFIR as a means by which to see far; a road-map for the journey of accumulating an ever more rich understanding of the complexities of implementation, and a more predictable means by which to ensure effective implementations.

## Competing interests

The authors declare that they have no competing interests.

## Authors' contributions

LJD and JCL conceived of the paper. LJD drafted the initial form and all revisions of this paper. All other authors (JCL, REK, DCA, SRK, JAA) made significant contributions to the conceptual framework and read and modified drafts. All authors read and approved the final manuscript.

## Supplementary Material

Additional file 1 **CFIR Figure and Explanatory Text**. This file provides a visual figure showing the main domains in the CFIR along with explanatory text. Click here for file

Additional file 2 **Matrix of Constructs from Models in the Literature to CFIR Constructs**. A matrix that shows a mapping of constructs from published theories to constructs included in the CFIR. Click here for file

Additional file 3 **CFIR Constructs with Short Definitions**. A 2-page table of CFIR constructs with short definitions that can be used as a quick reference. Click here for file

Additional file 4 **Detailed Rationale for Constructs**. Documentation of further references to support inclusion/definitions of constructs included in the CFIR. Click here for file
