# Supplementary material for: Fostering implementation of health services research findings into practice: a consolidated framework for advancing implementation science
Source: Implement Sci. 2009 Aug 7;4:50. doi: 10.1186/1748-5908-4-50 (PMC2736161; doi:10.1186/1748-5908-4-50)
Supplement: Additional file 1 — CFIR Figure and Explanatory Text. This file provides a visual figure showing the main domains in the CFIR along with explanatory text. [file 1748-5908-4-50-S1.pdf]

CFIR
Consolidated Framework for Implementation Research

ADDITIONAL FILE 1

CFIR Figure and Explanatory Text

Damschroder LJ, Aron DC, Keith RE, Kirsh SR, Alexander JA, Lowery JC. Fostering implementation of health services research findings into practice: A consolidated framework for advancing implementation science.

Figure 1: Major Domains of the CFIR

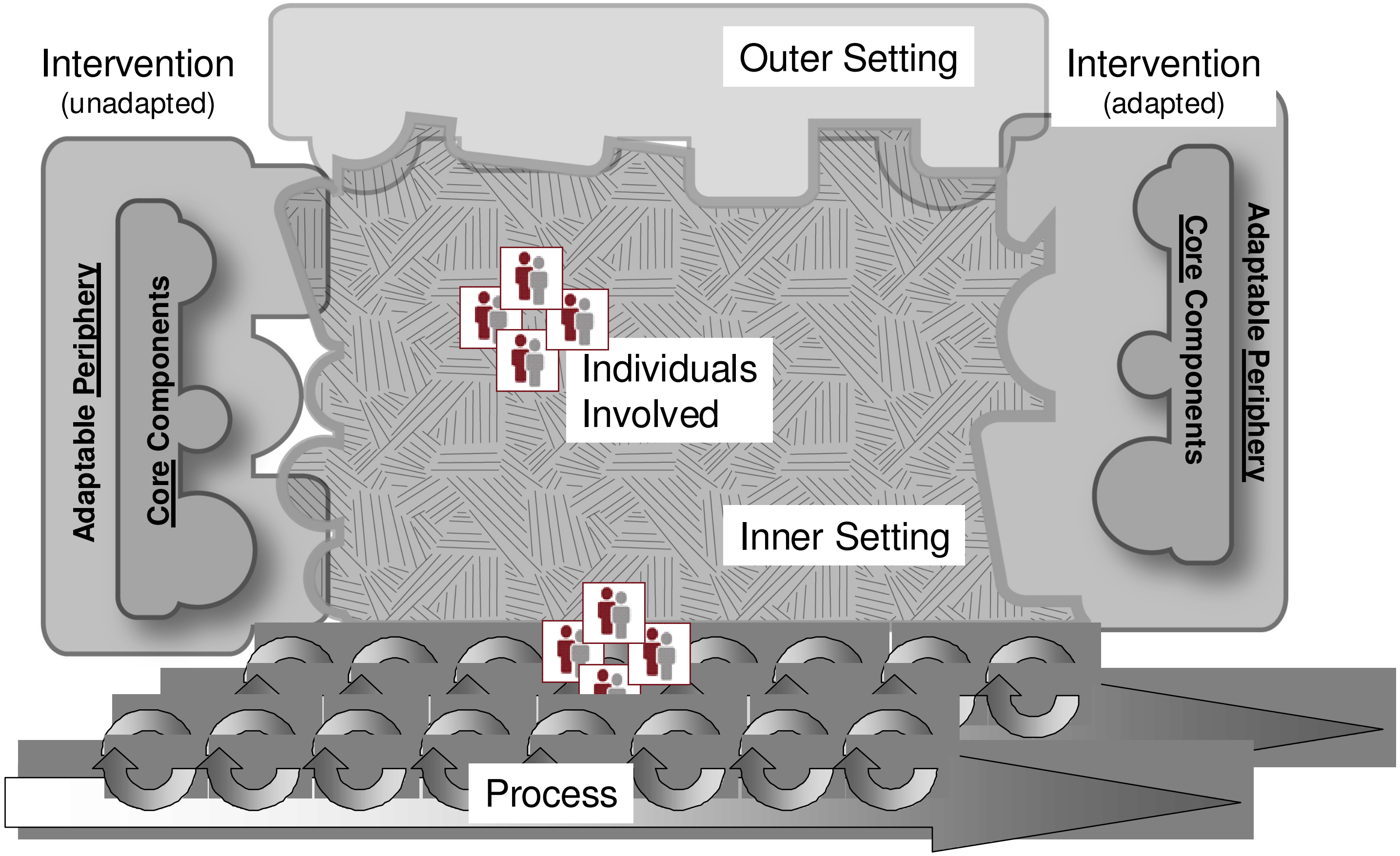

Figure 1 shows the CFIR's five major domains (the intervention, inner and outer setting, the individuals involved, and the process by which implementation is accomplished). The rather unconventional depiction used in Figure 1 conveys how these domains interact in rich and complex ways to influence implementation effectiveness. CFIR's domains were influenced by Pettigrew and Whipp, who more than 20 years ago, emphasized the essential interactive dimensions of content of intervention, context (inner and outer settings), and process of implementation [26]. This basic structure is also partially echoed by the PARiHS framework which describes the three key domains of evidence, context, and facilitation [14, 15]. Fixsen, et al emphasize the multi-level influences on implementation from external influencers to organizational and core implementation process components which include the central role of the individuals who coach and train prospective practitioners and the practitioners themselves [16]. The CFIR has added a domain for the individuals involved to acknowledge their important role in implementation.

The first major domain of the CFIR is related to characteristics of the intervention being implemented into a particular organization. Without adaptation, interventions usually come to a setting as a poor fit, resisted by individuals who will be affected by the intervention, and requiring an active process to engage individuals in order to accomplish implementation. The left side of Figure 1 shows an intervention that has not been adapted to the setting – the puzzle metaphor implied by the limits of our 2-dimensional diagram is simplistic. Imagining a cell and an external organism that must shift and reshape receptors to conjoin, though still incomplete, is an appropriate metaphor for the mutual shaping (co-evolution) that often occurs in both setting and intervention as implementation progresses [27]. The intervention is shown as an irregular shape because interventions are often complex, multi-faceted, and have many interacting components. A simple intervention might be visualized as a simple recognizable shape that is relatively straight-forward to implement. Interventions can be conceptualized as having core components (the essential and indispensible elements of the intervention itself) and an adaptable periphery (adaptable elements, structures and systems related to the intervention and organization into which it is being implemented) [13, 16, 28]. For example, a clinical reminder to screen for obesity must include an alert that pops up on the computer screen at the appropriate time for the appropriate patient. This feature is part of the "core" of the intervention. Just

as importantly, the intervention's "adaptable periphery" allows it to be modified to the setting without undermining integrity of that intervention. For example, depending on the work processes at individual clinics, the clinical reminder could pop up during the patient assessment by a nurse case manager or when the primary care provider evaluates the patient. Figure 1 delineates the intervention's core components and adaptable periphery. It shows visually that components of the periphery can be modified to a particular setting and vice versa in a co-evolving/co-adaptive way [29-31], moving from the left (un-adapted intervention and setting) to the right side of the Figure, where both the intervention and setting have changed shapes showing how they evolved or adapted to each other.

The next two domains in the CFIR are inner and outer setting. Most healthcare organizations are hierarchically organized and have interrelationships within and between other organizations (e.g., between outpatient clinics and a community hospital). Changes in the outer setting can influence implementation, often mediated through changes in the inner setting [32]. Generally, the outer setting includes the economic, political, and social context within which an organization resides and the inner setting includes features of structural, political, and cultural contexts through which the implementation process will proceed [33]. However, the line between inner and outer setting is not always clear and the interface is dynamic and sometimes precarious. This relationship is reflected by the overlapping, irregular, and thick grayed lines between inner and outer setting in Figure 1. The specific factors considered "in" or "out" will depend on the context of the implementation effort. For example, outlying clinics may be part of the outer setting in one study but part of the inner setting in another study. The inner setting is oddly shaped because of the complexities inherent to organizations comprised of tightly or loosely coupled entities (e.g., a medical center and outlying contracted clinics or integrated service lines within a health system). The inner setting is textured with hash lines to convey the tangible and intangible manifestation of structural characteristics, networks and communications, culture, climate, and readiness that all interrelate and influence implementation.

The fourth major domain of the CFIR is the individuals involved with the intervention and/or implementation process. Individuals have agency; they make choices and can wield power and influence on others with predictable or unpredictable consequences for implementation. Individuals are

carriers of cultural, organizational, professional, and individual mindsets, norms, interests, and affiliations. Greenhalgh et al describe the significant role of individuals:

> People are not passive recipients of innovations. Rather….they seek innovations, experiment with them, evaluate them, find (or fail to find) meaning in them, develop feelings (positive or negative) about them, challenge them, worry about them, complain about them, “work around” them, gain experience with them, modify them to fit particular tasks, and try to improve or redesign them – often through dialogue with other users.[13](p 598)

Many theories of individual change have been published [7] but little research has been done to gain understanding of the dynamic interplay between individuals and the organization within which they work and how that interplay influences individual or organizational behavior change. One recent synthesis of 76 studies using social cognitive theories of behavior change found that the Theory of Planned Behavior (TPB) model was the most often used model to explain intention and predict clinical behavior of health professionals. The TPB, overall, succeeded in explaining 31% of variance in behavior [34]. The authors suggest that “special care” is needed to better define (and understand) *the context* of behavior performance. Frambach and Schillewaert’s multi-level framework is unique in explicitly acknowledging the multi-level nature of change by integrating individual behavior change within the context of organizational change [35]. Figure 1 shows individuals in the inner setting who include both targeted users and other potentially affected individuals. Individuals are also depicted as part of the process. These individuals actively promote the implementation process and may come from the inner or outer setting (e.g., local champions, external change agents) and are thus, overlapping the two domains.

The fifth major domain is implementation process. Successful implementation usually requires an active change process aimed to achieve individual and organizational level use of the intervention, as designed. The implementation process is depicted in Figure 1 as an interrelated series of sub-processes that do not necessarily occur sequentially. The multiple series of cycles and shadowed arrows represent the complexity of executing and evaluating implementation because there are often related processes happening simultaneously at multiple levels within the organization [33]. These sub-

processes may be formally planned or spontaneous; conscious or sub-conscious; linear or non-linear but, ideally, are all aimed in the same general direction: effective implementation.

In summary, the CFIR's overarching structure supports the exploration of essential questions encountered in a study or evaluation of implementation (formative evaluations) [3, 27]. Using the five major domains as an initial organizing structure (i.e., intervention, outer and inner setting, individuals involved, and process), we consolidate the plethora of constructs described in Greenhalgh, et al's conceptual model and 18 additional models listed in Table 1. We combined some constructs within and across frameworks that have different labels but were redundant or difficult to distinguish from one another, and parsed apart constructs that conflated concepts. In some cases, models acknowledge the importance of, for example, setting but do not explicate specific features (e.g., Fixsen's implementation framework [16]). We provide detailed rationale for the constructs that constitute the CFIR in Appendix 1. The next section describes a formative evaluation study in which the CFIR was used to explore barriers and facilitators for uptake of a nationally disseminated program.
