# Supplementary material for: Fostering implementation of health services research findings into practice: a consolidated framework for advancing implementation science
Source: Implement Sci. 2009 Aug 7;4:50. doi: 10.1186/1748-5908-4-50 (PMC2736161; doi:10.1186/1748-5908-4-50)
Supplement: Additional file 2 — Matrix of Constructs from Models in the Literature to CFIR Constructs. A matrix that shows a mapping of constructs from published theories to constructs included in the CFIR. [file 1748-5908-4-50-S2.pdf]

CONTEXT

ADDITIONAL FILE 2: Matrix of Constructs from Models in the Literature to CFIR Constructs

Damschroder LJ, Aron DC, Keith RE, Kirsh SR, Alexander JA, Lowery JC:**Fostering implementation of health services research findings into practice: A consolidated framework for advancing implementation science**

Code	Topic/Description	1 Greenhalgh et al. [13]	2 Klein, Conn & Sorra [10,47]	3 Pettigrew & Whipp [26]	4 Leeman [44]	5 PARIHS Model [14,15]	6 Ottowa Model [50]	7 Simpson [70]	8 Kochevar & Yano [46]	9 Stetler [39]	10 Edmondson et al. [19]	11 Kilbourne et al.	12 VanDeusen Lukas et al. [80]	13 Grol, et al [7,106]	14 Mendel et al. [27]	15 Fixsen et al [16]	16 Brach et al. [99]	17 Glisson et al. [100]	18 Feldstein & Glasgow [43]	19 Frambach & Schillewaert [35]
	See Table 1 in main paper for full citations:	1	2	3	4	5	6	7	8	9	10	11	12	13	14	15	16	17	18	19
**I. INTERVENTION CHARACTERISTICS**				√																
A	Intervention Source									√	√			√						
B	Evidence Strength & Quality				√	√	√			√		√					√		√	
C	Relative advantage	√				√				√	√			√	√		√		√	√
D	Adaptability	√	√	√	√	√				√		√		√			√		√	
E	Trialability	√	√		√	√				√		√		√					√	√
F	Complexity	√	√						√	√	√			√	√		√		√	√
G	Design Quality and Packaging		√				√					√		√						
H	Cost						√					√		√	√		√		√	
**II. OUTER SETTING**				√						√					√	√				
A	Patient Needs & Resources					√	√					√	√		√			√	√	
B	Cosmopolitanism	√		√				√							√			√		√
C	Peer Pressure	√		√								√	√		√				√	√
D	External Policies & Incentives	√		√									√		√		√		√	
**III. INNER SETTING**				√						√		√			√	√				
A	Structural Characteristics	√		√											√					√
B	Networks & Communications	√	√	√		√	√	√					√		√		√	√	√	√
C	Culture	√	√	√		√	√	√					√		√			√	√	
D	Implementation Climate			√											√			√		√
1	Tension for Change	√		√						√		√	√				√			
2	Compatibility	√	√					√	√	√	√			√	√		√		√	√
3	Relative Priority		√							√					√				√	
4	Organizational Incentives & Rewards		√	√	√							√	√		√				√	
5	Goals and Feedback	√		√		√		√	√			√	√		√				√	
6	Learning Climate	√	√			√		√					√						√	
D	Readiness for Implementation			√															√	
1	Leadership Engagement	√	√	√	√	√		√		√	√		√				√		√	√
2	Available Resources	√	√	√		√		√		√	√		√		√		√			
3	Access to knowledge and information	√	√		√		√		√			√					√		√	√
**IV. CHARACTERISTICS OF INDIVIDUALS**				√			√													
A	Knowledge & Beliefs about the Intervention	√											√	√	√		√		√	√
B	Self-efficacy	√	√										√	√	√				√	
C	Individual Stage of Change	√	√											√	√				√	
D	Individual Identification with Organization														√		√	√		
E	Other Personal Attributes												√	√						√
**V. PROCESS**				√		√														
A	Planning	√		√						√		√		√	√	√	√	√	√	
B	Engaging	√		√						√	√	√	√	√	√	√		√	√	
1	Opinion Leaders	√				√					√	√			√		√		√	√
2	Formally appointed internal implementation leaders			√												√	√	√	√	
3	Champions	√											√				√		√	
4	External Change Agents	√													√		√	√	√	
C	Executing	√		√						√		√		√	√				√	
D	Reflecting & Evaluating	√		√						√	√	√		√	√	√	√	√	√	
