# Supplementary material for: Fostering implementation of health services research findings into practice: a consolidated framework for advancing implementation science
Source: Implement Sci. 2009 Aug 7;4:50. doi: 10.1186/1748-5908-4-50 (PMC2736161; doi:10.1186/1748-5908-4-50)
Supplement: Additional file 3 — CFIR Constructs with Short Definitions. A 2-page table of CFIR constructs with short definitions that can be used as a quick reference. [file 1748-5908-4-50-S3.pdf]

ADDITIONAL FILE 3: CFIR Constructs with Short Definitions

Damschroder LJ, Aron DC, Keith RE, Kirsh SR, Alexander JA, Lowery JC: **Fostering implementation of health services research findings into practice: A consolidated framework for advancing implementation science**

	Topic/Description	Short Description
**I. INTERVENTION CHARACTERISTICS**		
A	Intervention Source	Perception of key stakeholders about whether the intervention is externally or internally developed.
B	Evidence Strength & Quality	Stakeholders' perceptions of the quality and validity of evidence supporting the belief that the intervention will have desired outcomes.
C	Relative advantage	Stakeholders' perception of the advantage of implementing the intervention versus an alternative solution.
D	Adaptability	The degree to which an intervention can be adapted, tailored, refined, or reinvented to meet local needs.
E	Trialability	The ability to test the intervention on a small scale in the organization [8], and to be able to reverse course (undo implementation) if warranted.
F	Complexity	Perceived difficulty of implementation, reflected by duration, scope, radicalness, disruptiveness, centrality, and intricacy and number of steps required to implement
G	Design Quality and Packaging	Perceived excellence in how the intervention is bundled, presented, and assembled
H	Cost	Costs of the intervention and costs associated with implementing that intervention including investment, supply, and opportunity costs.
**II. OUTER SETTING**		
A	Patient Needs & Resources	The extent to which patient needs, as well as barriers and facilitators to meet those needs are accurately known and prioritized by the organization.
B	Cosmopolitanism	The degree to which an organization is networked with other external organizations.
C	Peer Pressure	Mimetic or competitive pressure to implement an intervention; typically because most or other key peer or competing organizations have already implemented or in a bid for a competitive edge.
D	External Policy & Incentives	A broad construct that includes external strategies to spread interventions including policy and regulations (governmental or other central entity), external mandates, recommendations and guidelines, pay-for-performance, collaboratives, and public or benchmark reporting.
**III. INNER SETTING**		
A	Structural Characteristics	The social architecture, age, maturity, and size of an organization.
B	Networks & Communications	The nature and quality of webs of social networks and the nature and quality of formal and informal communications within an organization.
C	Culture	Norms, values, and basic assumptions of a given organization.
D	Implementation Climate	The absorptive capacity for change, shared receptivity of involved individuals to an intervention and the extent to which use of that intervention will be rewarded, supported, and expected within their organization.
1	Tension for Change	The degree to which stakeholders perceive the current situation as intolerable or needing change.
2	Compatibility	The degree of tangible fit between meaning and values attached to the intervention by involved individuals, how those align with individuals' own norms, values, and perceived risks and needs, and how the intervention fits with existing workflows and systems.
3	Relative Priority	Individuals' shared perception of the importance of the implementation within the organization.
4	Organizational Incentives & Rewards	Extrinsic incentives such as goal-sharing awards, performance reviews, promotions, and raises in salary and less tangible incentives such as increased stature or respect.

5	Goals and Feedback	The degree to which goals are clearly communicated, acted upon, and fed back to staff and alignment of that feedback with goals.
6	Learning Climate	A climate in which: a) leaders express their own fallibility and need for team members' assistance and input; b) team members feel that they are essential, valued, and knowledgeable partners in the change process; c) individuals feel psychologically safe to try new methods; and d) there is sufficient time and space for reflective thinking and evaluation.
E	Readiness for Implementation	Tangible and immediate indicators of organizational commitment to its decision to implement an intervention.
1	Leadership Engagement	Commitment, involvement, and accountability of leaders and managers with the implementation.
2	Available Resources	The level of resources dedicated for implementation and on-going operations including money, training, education, physical space, and time.
3	Access to knowledge and information	Ease of access to digestible information and knowledge about the intervention and how to incorporate it into work tasks.
**IV. CHARACTERISTICS OF INDIVIDUALS**		
A	Knowledge & Beliefs about the Intervention	Individuals' attitudes toward and value placed on the intervention as well as familiarity with facts, truths, and principles related to the intervention.
B	Self-efficacy	Individual belief in their own capabilities to execute courses of action to achieve implementation goals.
C	Individual Stage of Change	Characterization of the phase an individual is in, as he or she progresses toward skilled, enthusiastic, and sustained use of the intervention.
D	Individual Identification with Organization	A broad construct related to how individuals perceive the organization and their relationship and degree of commitment with that organization.
E	Other Personal Attributes	A broad construct to include other personal traits such as tolerance of ambiguity, intellectual ability, motivation, values, competence, capacity, and learning style.
**V. PROCESS**		
A	Planning	The degree to which a scheme or method of behavior and tasks for implementing an intervention are developed in advance and the quality of those schemes or methods.
B	Engaging	Attracting and involving appropriate individuals in the implementation and use of the intervention through a combined strategy of social marketing, education, role modeling, training, and other similar activities.
1	Opinion Leaders	Individuals in an organization who have formal or informal influence on the attitudes and beliefs of their colleagues with respect to implementing the intervention
2	Formally appointed internal implementation leaders	Individuals from within the organization who have been formally appointed with responsibility for implementing an intervention as coordinator, project manager, team leader, or other similar role.
3	Champions	"Individuals who dedicate themselves to supporting, marketing, and 'driving through' an [implementation]" [101](p. 182), overcoming indifference or resistance that the intervention may provoke in an organization.
4	External Change Agents	Individuals who are affiliated with an outside entity who formally influence or facilitate intervention decisions in a desirable direction.
C	Executing	Carrying out or accomplishing the implementation according to plan.
D	Reflecting & Evaluating	Quantitative and qualitative feedback about the progress and quality of implementation accompanied with regular personal and team debriefing about progress and experience.
