# Supplementary material for: Fostering implementation of health services research findings into practice: a consolidated framework for advancing implementation science
Source: Implement Sci. 2009 Aug 7;4:50. doi: 10.1186/1748-5908-4-50 (PMC2736161; doi:10.1186/1748-5908-4-50)
Supplement: Additional file 4 — Detailed Rationale for Constructs. Documentation of further references to support inclusion/definitions of constructs included in the CFIR. [file 1748-5908-4-50-S4.pdf]

CFIR
Consolidated Framework for Implementation Research

ADDITIONAL FILE 4

Detailed Rationale for Constructs

Damschroder LJ, Aron DC, Keith RE, Kirsh SR, Alexander JA, Lowery JC. Fostering implementation of health services research findings into practice: A consolidated framework for advancing implementation science

INTRODUCTION

As stated in the main paper, the CFIR used Greenhalgh et al's synthesis [1] as a starting point, building on their work through review of 17 other models that were not explicitly included in their review. The CFIR focuses on constructs related specifically to implementation and subsequent routinization. Greenhalgh et al describe constructs related to diffusion and dissemination, system antecedents for innovation, system readiness for innovation, and implementation and routinization. They describe evidence about influences on implementation as "particularly complex and relatively sparse…" (page 610) and admit that many of the factors covered in the other domains also apply to implementation [1]. We combined applicable constructs from all domains for inclusion in the CFIR. Appendix 2 contains a matrix showing all the models included in the CFIR and their associated constructs, mapped onto CFIR constructs.

The remainder of Appendix 1 provides more detail about theoretical and empirical support for each construct. In most cases, we do not reiterate what is already included in the other models (as depicted in Appendix 2) – for example, nine of the 19 models include complexity as a significant influence on effective implementation but generally, we do not reiterate this fact for each construct. We do, however, provide rationale for significant departures in terminology or definitions embraced by other models.

I. Intervention Characteristics

Key attributes of interventions influence the success of implementation [1, 2]. There is broad support for this in the literature across many scientific disciplines.

A. Intervention Source -- Perception of key stakeholders about whether the intervention is externally or internally developed may influence success of implementation [1, 3]. The intervention may enter into the organization through an external source such as a formal research entity or vendor (see also, External Policy & Incentives under Outer Setting). Alternatively, an intervention may have been internally developed as a good idea, solution to a problem, or other grass-roots effort.

An externally developed intervention coupled with lack of transparency in the decision-making process may lead to implementation failure [4, 5]. Dissemination, “whose *main* mechanism of spread is centrally driven and controlled” is negatively associated with implementation ([1], page 604; emphasis added). Though there is empirical evidence of a positive association with an authoritative decision to use the intervention, there is a negative relationship with fully implementing or routinizing the intervention [1]. On the other hand, key ideas that come from outside the organization and that are then effectively tailored to the organization can result in successful implementation [6] (See Adaptability). Sometimes, internal decisions to adopt or implement an intervention may be perceived as externally driven. If the decision to adopt and implement is made by leaders higher in the hierarchy who edict change with little user input in the decision to implement an intervention, implementation is less likely to be effective [7, 8].

B. Evidence Strength and Quality -- Stakeholders’ perceptions of the quality and validity of evidence supporting the belief that the intervention will have desired outcomes.

Though there is no agreed upon measure of “strong evidence”, there is empirical support of a positive association with dissemination [9]. Support for the role of evidence strength and quality in implementation is mixed, however [9]. Though strong evidence is important, it is not always dominant in individual decisions to adopt nor is it ever sufficient [10]. Evidence supporting the use of an intervention may come through external sources (e.g., peer reviewed literature) or internally from other sources that appear to be credible [11]. The PARiHS model lists three sources of evidence as being key for research uptake: research studies, clinical experience, and patient experience [12]. External and internal evidence, including experience through piloting (see Trialability), may be combined to build a case for implementing an intervention [11]. The more sources of evidence used, the more likely innovations will be taken up [4, 5]. Credibility of the developers of evidence, transparency of the process used to develop (see Engaging), and intentionally mapping out the implementation (see Panning) to counterbalance negative and positive perceptions of the intervention by potential users are all important for effective implementation [13].

C. Relative advantage – Stakeholders’ perception of the advantage of implementing the intervention versus an alternative solution [6].

Relative advantage must be recognized and acknowledged by all key stakeholders for

effective implementation [1]. If users perceive a clear, unambiguous advantage in effectiveness or efficiency of the intervention, it is more likely the implementation will be successful. In fact, this perceived relative advantage is sine qua non for adoption/implementation [1]. Greenhalgh et al, the PRISM model, and Grol et al's implementation model all list observability as a separate construct [1, 14, 15]. Benefits of the intervention must be clearly visible (observable) to stakeholders to assess relative advantage and thus, efforts to demonstrate benefits of the intervention clearly will help implementation [1, 9, 14, 16, 17]. Observability was incorporated into the relative advantage construct. Observability (or visibility) of benefits is tightly coupled with stakeholders' perception of relative advantage and it would be challenging to tease out separable measures for both in the real world. Thus, we consolidated the two factors, while acknowledging the role of both. The extent to which the intervention is codifiable may also influence perception of relative advantage. Many interventions contain significant tacit components and may have significant benefits that are more difficult to understand or discern [18, 19] and thus evaluate for relative advantage.

D. Adaptability – The degree to which an intervention can be adapted, tailored, refined, or reinvented to meet local needs. Adaptability relies on a definition of the intervention's "hard core" (the "irreducible" elements of the intervention itself) versus "soft periphery" (adaptable elements, structures and systems related to the intervention and organization into which it is being implemented) [1, 16] of the intervention.

Information about the hard core and soft periphery can be used to assess "fidelity" [16]. The hard core may be defined by a research protocol or "black-box" packaging while the soft periphery consists of factors that vary from site to site. For example, a computerized report system has a fundamental core that users cannot change but it might be accessed from different launch points, depending on workflows of individual organizations. Greenhalgh, et al describe aspects of adaptability under "fuzzy boundaries" and "potential for reinvention" [1] (page 596-597). There is a natural tension between the need to achieve full and consistent implementation across multiple settings while providing the flexibility for local sites to adapt the intervention as needed [20]. An intervention that can be easily modified to adapt to the setting is positively associated with implementation [6, 21, 22].

E. Trialability – The ability to test the intervention on a small scale in their own organization [1] and reverse course (undo implementation) if warranted [15].

The ability to pilot an intervention has a strong positive association with effective implementation [1]. Based on trial results, the organization may decide to go forward with full implementation or retool and modify as needed. Also, a trial will provide needed information about how best to implement to other units to minimize workflow disruption. Pilot testing is a key feature of the Plan-Do-Study-Act quality improvement cycle which allows users to find ways to increase coordination to manage interdependence [22]. Piloting allows individuals and groups to build experience and expertise, and time to reflect upon and test the intervention [4, 5]. Users need to be able to stop the intervention and reverse its effects if it causes problems or is ineffective [15].

F. Complexity – Perceived difficulty of implementation, reflected by intervention type (e.g., behavior change or plug-in technology), duration, scope, radicalness,

disruptiveness, centrality, intricacy, and number of steps required to implement [1, 6, 14].

We have combined several factors that are closely intertwined with stakeholders' perception of complexity: the pervasiveness, scope, impact, radicalness, magnitude, disruptiveness, centrality and duration of the intervention [1, 14]. Radical interventions require reorientation and non-routine processes that produce fundamental changes in the organization's activities and a clear departure from existing practices [1] as do interventions affecting core work processes [14]. Appropriately diagnosing and assessing complexity is thought to benefit implementation by avoiding unintended consequences [23]. There is a negative association between stakeholder's perception of how complex an intervention is and effective implementation (i.e., simple interventions are more likely to be effective) [1, 6] because it affects user satisfaction and the speed required to be competent in using the interventions [7]. One aspect of complexity can be determnined by assessing the "length" (the number of sequential subprocesses or steps for using or implementing an intervention) and "breadth" (number of choices presented at decision points) [23]. Complexity is increased when the intervention is targeting higher numbers of potential target organizational units (teams, clinics, departments) or types of people (providers, patients, managers) [23]. The ability to implement an intervention incrementally (sometimes referred to as divisibility [14]; see Executing) can influence perception of complexity.

The type of intervention, whether a technical (e.g., a new computer module) or an administrative change (behavioral change), can contribute to the perception of complexity. Technical interventions may include a purchased product, packaged service, or an automated production process (e.g., computerized order entry). Administrative interventions primarily affect organizational social structures or processes. Most interventions are a hybrid of both. Technical interventions tend to be more visible and administrative interventions tend to be more complex and difficult to implement [1]. On the other hand, complex behavioral change interventions can also work in favor of implementation. If organizations embrace an intervention as a fundamental change to processes up front, they are more likely to do what it takes to fully and effectively implement the intervention compared to sites that regard it as a simple "plug-in" intervention [24]. Edmondson and colleagues describe a "technological frame" of thinking that influences implementation effectiveness. In their study of a new cardiac surgical approach, the sites with less successful implementation viewed the intervention as a "plug-in technology" while those with better implementation effectiveness regarded the intervention "as fundamental change for the [operating] team" [24] and had more engaged support by key stakeholders (see Leadership Engagement and Engaging). Regardless of the degree of complexity, simple, clear, and detailed implementation plans, schedules, and task assignments contribute to successful implementation [6].

<u>G. Design Quality and Packaging</u> – Excellence in how the intervention is bundled, presented, and assembled [7].

This construct was not included in Greenhalgh, et al's model but is included in Grol and Wensing's list of innovation characteristics [14]. Packaging is related to how the intervention is bundled, presented, or assembled and even how accessible it is for users. When components are more easily accessible to users, it promotes use of the

new procedures [13]. An unreliable or imperfectly designed intervention will undermine success [7]. When intervention quality is perceived to be poor by users, there are negative consequences for employee satisfaction and intervention use [7, 8].

H. Cost -- Costs of the intervention and costs associated with implementing that intervention including investment, supply, and opportunity costs.

Costs of the intervention and those associated with implementing an intervention are negatively associated with implementation [13, 25].

II. Outer Setting

The influence of factors from the outer setting is clear [1]. Healthcare systems are hierarchically organized and are thus often interrelated. Changes in the outer setting can have positive or negative influences on implementation; often mediated through changes in the inner setting [26].

A. Patient needs & resources -- The extent to which patient needs, as well as barriers and facilitators to meet those needs are accurately known and integral to the organization.

Many models and theories of research uptake or implementation do acknowledge the importance of accounting for patient characteristics [13] and assume that if they are not accounted for, implementation will be less effective [4, 5]. The quality improvement literature has acknowledged that having a strong focus on the customer (patient) is an essential "core property" for making progress in implementing quality improvement initiatives [26, 27]. Organizations who use evidence-based practices more consistently relied on knowledge from nurses' experiences with patient interactions [28]. Effective implementation can be predicted, in part, by the extent to which organizations provide tools and advice that are matched to patients' readiness to change and when choices are presented to enhance patient activation and autonomy [15]. The extent to which an organization is successful in achieving this degree of "patient-centeredness" can be determined by assessing factors in the inner setting e.g., related to Goals and Feedback (the extent to which goals and feedback are aligned with patient needs) and Compatibility (the extent to which an intervention targeted to help patients aligns with staff values and meaning attached to the intervention). Attention on patient satisfaction is consistently associated with greater perceived team effectiveness which in turn, influences the number and depth of changes implemented in an organization [29].

B. Cosmopolitanism – the degree to which an organization is externally networked with other external organizations.

Informal inter-organizational networks demonstrate the degree to which an organization is externally networked which in turn, indicates the degree of cosmopolitanism [1]. Organizations that support and promote external boundary-spanning roles are more likely to implement new practices quickly [1, 30-33]. Active participation with professional group(s), keeping up with salient literature and research findings, updating skills, and providing opportunities for external training is associated with implementation [34]. Professional knowledge typically arises because of increased boundary spanning activities, self-confidence and commitment to move beyond status quo [35]. There is a negative relationship between cosmopolitanism

and implementation until clear advantages of the intervention become apparent [1, 36]. But the relationship is positive once the innovation is accepted as the norm by others in the in/formal network (see Peer Pressure) [1]. Greenhalgh et al call this external boundary spanning (internal boundary spanners are included under Network and Communications below) and describe some aspects of cosmopolitanism under informal interorganizational networks. When organizations promote and support external linkages with outside organizations through their staff, they will assimilate innovations quicker [1].

Several strands of research explore relationships inside and outside one's organization and apply to both Cosmopolitanism and Networks and Communications in the inner setting. Social capital is one term often used to describe the quality and the extent of social interactions. Dimensions of social capital include trust, shared vision, and information sharing. Social capital can be subdivided into 1) internal bonding social capital that examines behavior within a group of people within the same facility and 2) external bridging social capital that examines connections to or with people or groups outside the facility. Each individual's relationships with other individuals both within and outside of the organization represent that individual's social capital. In turn, the collective networks of relationships of the individuals in an organization represent the social capital of the organization [37-40].

C. Peer Pressure -- Mimetic or competitive pressure to implement an intervention; typically because most or other key peer or competing organizations have already implemented or in a bid for a competitive edge.

There is strong direct evidence that the pressure to adopt an intervention, independent of their perception of whether their patients need it or in response to a perceived problem, influences organizational adoption and implementation, particularly in highly cosmopolitan organizations [1]. In highly competitive markets, organizations may more likely to implement new interventions [41]. "Peers" can refer to any outside entity which with the organization feels some degree of affinity or competition with at some level within their organization (e.g., competitors in the market, other hospitals in a network, another highly regarded institution). If competitors or colleagues in other organizations or in other parts of the same organization are all using an innovation, people may feel compelled to do so as well. This is referred to as "mimetic pressure" or "inter-organizational norm-setting" [1]. This pressure directly influences adoption decisions but can also trickle down to implementation as well, if individual stakeholders are attuned to practices of outside entities or individuals. The pressure to adopt under these circumstances is particularly strong for late-adopting organizations [42].

D. External Policies & Incentives – A broad construct that includes external strategies to spread interventions including policy and regulations (governmental or other central entity), external mandates, recommendations and guidelines, pay-for-performance, collaboratives, and public or benchmark reporting [43].

We combined political directives into intentional spread strategies because sometimes directives are incorporated into collaboratives or other spread strategies. Greenhalgh, et al describe "political directives" and include "policy 'push'" and "external mandates" and cite evidence as strong (direct or indirect) in increasing motivation, though not capacity, of organizations to implement [1](page 610). Evidence for the influence of guidelines and public reporting is sparse and equivocal.

The threat or reality of public reporting may also motivate organizations, especially late-adopters, to implement an intervention in an effort not to look bad compared to their competitors. However, public reporting can also have a negative influence if there is an adversarial relationship between the reporting entity and the target organization. In this context, people may cover-up (compliant implementation) [44] or engage in "box-checking" rather than true committed use. Membership in a collaborative which adds a more active component to semi-public reporting and guidelines may foster successful implementation but formal quality improvement collaboratives have mixed effects [1]. Typically, collaboratives provide the opportunity to benchmark against peers in conjunction with implementing new practices or products. Reimbursement systems and other incentives (usually monetary) that are controlled outside the organization are another external force that can influence implementation [14].

III. Inner Setting

The inner setting is increasingly recognized as an active interacting facet and not just as a backdrop in implementation [45]. Every model we reviewed considered aspects of inner setting as shown in Appendix 2.

A. Structural Characteristics – the social architecture that describes how large numbers of people are clustered into smaller groups and differentiated and how the independent actions of these differentiated groups are coordinated to produce a holistic product or service [46, 47]. This construct also includes characteristics of organizations such as age and size.

Much support for the role of structural characteristics comes out of Damanpour's seminal research into organizational innovation [35]. Organizational innovation includes implementation of innovations along with generating and developing innovations. We extend findings into implementation as one key component of organizational innovation. Several dimensions of structural characteristics have been found to have significant associations with implementation. Many of these characteristics, however, have shown mixed effects most likely because they interact with other features of the organization [41].

Functional differentiation is the internal division of labor where coalitions of professionals are formed into differentiated units. The number of units/departments represents diversity of knowledge in an organization. The degree of specialization (the number of different occupational types or specialties in an organization) can have a positive relationship with implementing change when the knowledge base is increased [31]. The number of departments that participate in decision-making is positively associated with effective implementation [1, 32, 33, 35]. Centralization (the dispersion or concentration of decision-making autonomy) has mixed effects depending on the study [35] and the stage of intervention (initiative stage v. implementation stage) [48]. The degree of vertical integration (the number of hierarchical levels in departments or units) has a mixed relationship with implementation [32, 35, 49]. Administrative intensity (the ratio of managers to total employees) has a positive relationship with implementation [35].

Organization size is sometimes used as a proxy for other structural determinants. Size and age of an organization are both negatively associated with implementation when bureaucratic structure is increased as a result [42, 50].

B. Networks and Communications – The nature and quality of webs of social networks and the nature and quality of formal and informal communications within an organization.

The network and communications construct in the CFIR consolidates several domains from Greenhalgh, et al including organizational structure, intraorganizational communication, and intraorganizational networks [1]. Little is known about the interplay between formal structure, informal networks, and effective communication. Thus, we consolidate these concepts into a single construct to give wide latitude for researchers to take deductive approaches to explore alternate theories such as social networking [51] or complexity [52, 53] theories. This construct, perhaps more than any other, requires a more grounded approach of study; leaning toward a constructivist approach for analysis [54] until we understand more about the role of networks and communications and especially how it interrelates with other constructs.

Greenhalgh et al cite strong or moderate influence of intraorganizational communication, intraorganizational networks, internal boundary spanning, and organizational structure on implementation [1]. Coordination across departments and specialties is essential for effective implementation to attenuate the "complex web of sources of power and covert and overt sources of influence" that all contribute to individual decisions about whether to cooperate [15](p 233). A core principle of complexity theories leads to the idea that the actions of individuals and units affect implementation, positively or negatively, in predicted and unpredicted ways [55]. Integration to bridge traditional intra-organizational boundaries among individual components is one of five critical elements for driving transformation in patient care [56]. Connections between individuals, units, services, hierarchies may be strong or weak, formal or informal, visible or invisible. Physicians and nurses may struggle with routine (and role) changes that require coordination of activities and sharing of information across professions or units [7]. Clear role definitions (e.g., physician and non-physician roles) positively influences implementation [57, 58]. The PARHiS model asserts that clearly defined physical, social, cultural, structural, and system boundaries contributes to research uptake [4, 5].

Teamwork is emphasized as an essential "core property" for successful implementation of quality improvement initiatives [27](page 287). The more stable teams are (members are able to be with the team for an adequate period of time; low turnover), the more likely implementation will be successful [24]. Relationships with others, teamwork, and power and authority structures are all part of network and communications and influence implementation [4, 5].

Regardless of how an organization is networked (formally and informally), the importance of communication across the organization is clear. Communication failures are involved with the majority of sentinel events in US hospitals – most often between physicians and nurses [59]. High quality formal communications contribute to effective implementation [34]. Making staff feel welcome (good assimilation); peer collaboration and deprivatization and review (in the context of feedback about work practices from peers), clearly communicated mission and goals (see Goals and Feedback), cohesion between staff, informal communication quality, all contribute to implementation [34]. Strong horizontal and informal networks have positive relationship with adoption; e.g., physicians are influenced by the spreading of information through peers [1]. Strong intra-organizational communications are positively associated with implementation as is devolving decision-making to the

front-line teams or individuals [1, 60]. Strong communication includes having access to information (see Access to Information), being well-informed, and understanding the goals related to an intervention and all contribute to successful implementation [8]. Vertical and formal networks can also have a positive relationship with implementation; e.g., nurses may be most influenced by authoritative decisions [1].

C. Culture – Norms, values, and basic assumptions of a given organization [61]

Culture is not defined consistently in the literature [61] and is challenging to generalize its influence on implementation. We include it in the CFIR because, despite variation in use and definition, it has been shown to have significant influence on implementation effectiveness [62, 63].

Employees impart organizational culture to new members, and culture influences in large measure how employees relate to one another (see Networks and Communications) and their work environment [63]. Nearly all change efforts are targeted at visible, largely objective aspects of an organization that include work tasks, structures, and processes. One explanation for why so many of these initiatives fail, centers on the failure to change the less tangible organizational assumptions, thinking, or culture [64].

Theorists propose that organizational culture is among the most critical barriers to leveraging new knowledge and implementing technical interventions. Culture is an important component of the inner setting [4, 5]. It is measured by eliciting information from *individuals* – information can then be consolidated by *team or unit* and for the *organization* as a whole (though this is admittedly, a reductionist approach that falls short of describing how culture is defined through very fluid interlacings of people and networks at many levels). The next paragraph describes one measurement approach, the competing values framework (CVF) that has been used in healthcare (for example, [62]) and it has been used in the VA with mixed success [63]. However, we do not espouse any particular approach. We include a description of the CVF because of the frequency of its use in healthcare.

The CVF was originally developed by Quinn and Rohrbaugh [65] and is an example of a "variable definition" approach to culture: a quantitative measure that purports to capture key aspects of the complicated dynamics of culture. Often measures of culture are elicited from senior leaders in the organization – not from non-supervisors. The CVF characterizes organizations along two dimensions, each representing a basic challenge that every organization must resolve in order to function effectively. The first set of competing values is the degree to which an organization emphasizes central control over processes versus decentralization and flexibility. The second set of competing values is the trade-off between focus on its own internal environment and processes versus the external environment and relationships with outside entities. Four archetypical organizational cultures arise: 1) team culture (high internal focus with high flexibility (aka personal)); 2) hierarchical culture (high internal focus with high control (aka formalized and structured)); 3) entrepreneurial culture (high external focus with high flexibility (aka dynamic and entrepreneurial)); and 4) rational culture (high external focus with high control (aka production oriented)) [62, 63]. These "archetypes" are *not* mutually exclusive. In one study, CVF culture was not found to be influential in the number of evidence-based practices used by healthcare organizations [62]. However, organizational cohesion and adaptability to change are important [34], which are features found in

entrepreneurial-leaning organizations. Formalization is negatively associated with
innovation because of lack of flexibility and/or low acceptance of new ideas [35] and can foster continuance of status quo [7]. A "balanced" culture with respect to the
Competing Values Framework (how close organizations are to 25-25-25-25% on each of the four archetypical quadrants using a Herfindahl-type measure) contributes to perceptions of team effectiveness and in the number of changes implemented (though not depth of change) [29].

D. Implementation Climate -- The shared receptivity of involved individuals to an intervention [1] and the extent to which use of that intervention will be "rewarded, supported, and expected within their organization" [44](p 1060).

We introduce the concept of implementation climate, that is adapted from Klein and Sorra's conceptual model [44] which they also tested empirically [7, 8]. It is important to recognize that organizations have a multiplicity of coexisting cultures and climates. Greenhalgh, et al refer to "receptive context for change" and "absorptive capacity" which each include an amalgam of several factors. We parse these out separately, defining some as part of implementation climate and others as part of readiness for implementation. Implementation climate is a socially-constructed concept (i.e., a collective reflection of stakeholders' experience of culture [61] as it relates to a particular implementation) and may transcend vertical and horizontal organizational boundaries and interrelationships between them. However, climate may vary from unit to unit.

The appropriate scope of implementation climate (at the team, unit, service line, organization, system level, etc.) is determined by the scope and nature of

SIDEBAR

A discussion of readiness for change, receptive context, and implementation climate.

Models in the literature very widely in the use of these terms, their definitions and degree of specificity. Greenhalgh et al refer to "receptive context for change" and do not define it other than to say that it is associated with organizations "being better able to assimilate innovations" [1](p 607). Factors thought to contribute to receptive context are absorptive capacity for new knowledge, strong leadership, clear strategic vision, good managerial relations, visionary staff in pivotal positions, climate conducive to experimentation and risk-taking, and effective data capture systems. Greenhalgh et al also refer to "system readiness for innovation which refers to an organization's willingness "to assimilate a particular innovation" and factors contributing to system readiness include tension for change, innovation-system fit, assessment of implications (full assessment, ahead of time, of implications and subsequent effects), support advocacy, dedicated time and resources, and capacity to evaluate the innovation (p 608).

The PARiHS framework refers to readiness for change which is determined by receptivity for change, culture, leadership, and evaluation capability [5].

Lehman et al developed an Organizational Readiness for Change tool which is comprised of motivation for change (including tension for change), adequacy of resources, staff attributes, and organizational climate which includes mission, cohesion, autonomy, communication, stress, and change [66].

Klein, Conn, and Sorra introduce the concept of implementation climate[7] which is "targeted employees' shared summary perceptions of the extent to which their use of a specific innovation is rewarded, supported, and expected within their organization" and is comprised of ensuring skills through training, assistance, and enough time for implementation, providing incentives, removing obstacles (e.g., help by leadership, access to expertise). Antecedents for a positive implementation climate are adequate resources, management support, and implementation policy and procedures (the extent to which organizational policy and procedures support the implementation; e.g., training, quality and accessibility of the intervention, user support, time for experimentation, communications about the implementation).

These are just a few examples of the breadth of difference in how these terms are used. The CFIR includes implementation climate (extending Klein, Conn, and Sorra's definition) and Readiness for Implementation which is comprised of more immediate and tangible factors of available resources, leadership engagement (commitment), and infrastructure for information and knowledge to support intervention adoption and use.

the particular intervention. It is important to understand up front in a particular study, the organizational level at which "implementation climate" is salient. Implementation climate has a significant mediating role between management support and implementation effectiveness [7]. Implementation climate is composed of: tension for change, compatibility, relative priority, incentives and rewards, goals and feedback, and learning climate.

<u>D1. Tension for change</u> – The degree to which stakeholders perceive the current situation as intolerable or needing change [1, 34, 57, 58].

An acute sense of the need for change can be the trigger for designing an intervention internally. Externally developed interventions are often in response to a need for change (or gap in performance) at a macro level, rather than locally. Whether or not local stakeholders who are involved in local implementation actually feel a tension for change is an important antecedent for successful implementation [17, 56]. Effective communication (see Networks and Communications) can foster tension for change by building dissatisfaction with status quo as well as announcing a change, cultivating commitment, and reducing resistance [1]. When stakeholders have first-hand experience with the problem, implementation is especially more likely to be successful [6]. It is difficult to create a tension for change when none actually exists.

<u>D2. Compatibility</u> – The degree of tangible fit between meaning and values attached to the intervention by involved individuals, how those align with individuals' own norms, values, and perceived risks and needs, and how the intervention fits with existing workflows and systems [1, 44].

Greenhalgh et al describe compatibility, meaning, and innovation-system fit. All of three of these terms have a common root definition of aligning with stakeholders' values and norms and then vary in the degree to which they incorporate: goals, skill mix, strategies, and perceived needs [1]. We consolidate these together under our definition of compatibility. Greenhalgh et al cites strong indirect and moderate direct support for this somewhat elusive construct. For individuals, the perception of the degree of alignment between the meaning they attach to the intervention and meaning communicated by upper management has a strong influence on whether they will embrace the intervention [1]. For example, an intervention that leadership believes will improve performance may be perceived as a threat to autonomy in treatment decisions by physicians. Meaning in this context can be negotiated and reframed through discussions across organizational networks [1]. Compatibility is between the intervention and supporting technologies and ways of working [1] is also important. The degree of compatibility has a positive association with implementation [1, 44, 67]. Changes seen as threatening or incompatible with current conditions will be resisted [6]. Helfrich and colleagues found that perceived fit with organizational and professional mission, core competencies, and experience was an important predictor of successful implementation [8]. A balanced distribution of the risks and benefits throughout the organization increases the likelihood of acceptance [17].

<u>D3. Relative priority</u> – Individuals' shared perception of the importance of the implementation within the organization [7, 15, 44].

Few models explicitly incorporate the concept of relative priority. However, this construct has been found to be a significant predictor for implementation

effectiveness [7]. If employees perceive that implementation is a key organizational priority (promoted, supported, and cooperative behaviors rewarded), then implementation climate will be strong [7]. When relative priority is high, employees regard the intervention as an important priority rather than a distraction from their "real work" [7]. The higher the relative priority of implementing an intervention, the more effective the implementation is likely to be [7, 8]. The ability of an organization to fully implement may be a function of how many other initiatives or changes have been rolled out in the recent past which may lead to being overwhelmed with yet another initiative [1, 6] and a low priority being assigned.

D4. Organizational Incentives & Rewards – Extrinsic incentives such as goal-sharing awards, performance reviews, promotions, and raises in salary; also includes less tangible incentives such as increased stature or respect [7, 8].

Financial incentives and performance evaluations are important for reinforcing beliefs that behavior will lead to desirable results [6, 22]. Strong incentives were found to be an important component of implementation policy and practices and is positively associated with implementation effectiveness [7, 8]. A four-item "reward system" subscale is included as part of the Competing Values Framework measure of culture and it was found that the number of different types of compensation incentives used is positively associated with comprehensiveness of the use of best practices by healthcare organizations [62].

D5. Goals and Feedback – The degree to which goals are clearly communicated, acted upon, and fed back to staff and alignment of that feedback with goals [23, 34, 56, 68-70].

An important feature of many individual behavior change models is setting goals and receiving feedback on progress [68]. A recent review found that effects of using audit and feedback mechanisms to improve practices resulted in small to moderate effects [69]. However, studies showed wide variation of effects and little is known about appropriate methods. In addition, nothing was said about how those audit and feedback methods aligned with larger organizational mission and goals. Stakeholders cannot take corrective action without sufficient feedback that is tightly coupled with goals that are important to them [70]. Goal setting provides a benchmark that allows people to assess whether or not the intervention is creating value [69]. From an organizational perspective, the degree to which goals are clearly communicated, acted upon, and staff knowing what is measured, monitored, and reported is important to for successful implementation [56]. Less aggressive incremental goals [69] and goals that are specific and attainable [23] will increase implementation effectiveness. Leadership commitment, involvement, and accountability are key features for successful implementation (see Leadership Engagement). Among the most effective ways to engage that support is to have a change effort that is aligned with and contributes to achieving the organizational goals of leadership [56]. Support from leaders is more likely if they are kept involved in and informed about the implementation [17]. A sense of collective responsibility is thought to contribute to implementation success along with a communal understanding of mission and goals and the sense that task decisions are made based on them [34]. The Chronic Care Model emphasizes the importance of relying on multiple methods of evaluation and feedback including clinical, performance, economic evaluations, and experience [57, 58].

D6. Learning Climate – A climate in which: a) leaders express their own fallibility and need for team members' assistance and input; b) team members feel that they are essential, valued, and knowledgeable partners in the change process; c) individuals feel psychologically safe to try new methods; and d) there is sufficient time and space for reflective thinking and evaluation (in general, not just in a single implementation) [7, 44, 71].

We include learning "climate" instead of "culture" (as described in most of the literature) because we are interested in the learning climate of micro-environments related to specific implementations. The degree to which an organization demonstrates "learning" attributes will vary across sub-groups and local manifestations of these attributes may have a stronger influence than a measure of overall organizational learning [24]. The literature on team learning has emphasized the organization's role in creating the climate to enable learning and fosters collaboration within and between cross-disciplinary teams [72]. Quantitative measurement instruments are available for measuring an organization's "learning" capability [73, 74]. However, there is not agreement on precisely how to operationalize this construct. Despite this, we can make some generalizations. In a positive learning climate, stakeholders are not constrained by failure. A climate of psychological safety is promoted. Psychological safety has predicted engagement in quality improvement work [71]. Having the time and space for reflective thinking and evaluation (see Reflecting and Evaluating) is another important characteristic at least, in part, because it promotes learning from past successes and failures to inform future implementations [8, 24, 68]. Developing a climate that promotes learning is a "core property" that health care organizations need for on-going quality improvement [27](pg. 287). A learning climate is an important contributor for increasing absorptive capacity for new knowledge: the ability of an organization to fully assimilate interventions [1]. Greenhalgh, et al include learning organization culture within their concept of absorptive capacity of new knowledge along with existing knowledge and skills (tacit and explicit), and within proactive leadership.

E. Readiness for Implementation -- Tangible and immediate indicators of organizational commitment to its decision to implement an intervention, consisting of 3 sub-constructs. Implementation readiness is differentiated from implementation climate in the literature, by its inclusion of specific tangible and immediate indicators of organizational commitment to its decision to implement an intervention.

Implementation readiness is differentiated from implementation climate by its inclusion of more tangible and even more immediate indicators of organizational commitment to its decision to implement an intervention. The term "readiness for change" has a broad range of conceptualizations in the literature. Simpson and colleagues developed an organizational readiness for change measurement instrument that includes 18 organizational domains [75]. The PARiHS model describes readiness for change in terms of setting, culture, leadership, and evaluation [5, 76]. Greenhalgh, et al include tension for change, innovation-systems fit, assessing implications, support and advocacy, time and resources, and capacity to evaluate in their "system readiness for innovation" domain [1] (page 607-608). In addition, both the PARiHS model and Greenhalgh, et al describe "receptivity for change;" a related but not synonymous term. In the PARiHs model, it is included as a domain in the inner setting that indicates readiness for change while Greenhalgh, et al present "receptive context for change" as a general feature of organizations that include six over-arching constructs (e.g. strong leadership; page 607). We describe below, those constructs that contribute directly to an organization's

readiness for implementation: leadership engagement, available resources, and access to information and knowledge.

E1. Leadership Engagement – Commitment, involvement, and accountability of leaders and managers [7, 8, 17, 56, 77].

Commitment, involvement, and accountability of leaders and managers have a significant influence on the success of implementation [17, 56]. Anything less than wholehearted support from leaders dooms implementation to failure [77]. Leadership support in terms of commitment and active interest leads to a stronger implementation climate which is, in turn, related to implementation effectiveness; this association is strengthened, the more users are required to work together to implement [7, 8]. Leaders can be important conduits to help persuade stakeholders via interpersonal channels and by modeling norms (see Learning Climate) associated with implementing an intervention [22]. Managerial patience (taking a long-term view rather than short-term) allows time for the often inevitable reduction in productivity until the intervention takes hold which heightens the likelihood of successful implementation [7]. Middle managers, in addition to high-level leaders, are important for their ability to network (see Networks and Communication) and negotiate for resources (see Available Resources) and priority (see Relative Priority). Middle managers are more likely to support implementation if they believe that doing so will promote their own organizational goals (see Compatibility) and if they feel involved in discussions about the implementation (see Engaging) [60].

E2. Available Resources – The level of resources dedicated for implementation and on-going operations including money, training, education, physical space, and time [2, 16, 20, 22, 59, 60].

The level of resources dedicated for implementation is positively associated with implementation [2, 16, 20, 22, 59, 60] but is not necessarily sufficient to guarantee success [11]. Financial resources acts as a partial mediator between management support (see Leadership Engagement) and implementation policy and procedures [7]. Organizations may have "slack resources" that allow people to "squeeze" time for adopting an implementation on top of what they are already doing without noticeably impacting another area. Slack resources are thought to promote absorptive capacity because of the increased ability to absorb failure [35, 78]. Though, in a meta-analysis of several studies slack resources was not a significant influence, perhaps because of the lack of distinction between different types of slack resources [35].

E3. Access to information and knowledge – Ease of access to digestible information and knowledge about the intervention and how to incorporate it into work tasks [1, 7, 8, 79].

Ready access to digestible information about the intervention and how to incorporate it into work tasks is essential for successful implementation [1, 7, 8]. Information and knowledge includes all sources such as experts, staff with experience (including those not necessarily achieving "expert" status), training, and computerized information systems. The number of different knowledgeable occupational types or specialties who involved with the implementation is positively associated with effective implementation [79]. When timely on-the-job training is available, especially at a team level, implementation is more likely to be successful [1]. Education, training, and access to information about the intervention are all key

strategies to move stakeholders from unengaged to fully committed users of the intervention [14].

IV. Characteristics of Individuals

Organizations are made up of individuals. Setting and intervention constructs are rooted, ultimately, in the actions and behaviors of individuals. Little is known about the interplay between individuals and their ripple effects through their teams, units, networks, and organizations on implementation.

A. Knowledge and Beliefs – Individuals' attitudes toward the intervention and familiarity with facts, truths, and principles related to the intervention.

Users' *skilled* and *enthusiastic* use of the intervention, are two key features of effective implementation [44]. These attributes are important to understand at individual and sub-group levels in order to assess quality of implementation and prospects for sustainability.

Skill in using the intervention is a primarily cognitive function that relies on adequate how-to knowledge and knowledge of underlying principles or rationale for adopting the intervention [21]. Skill levels reflect the effectiveness of training. If this knowledge (see Access to Information and Knowledge) is not obtained prior to an individual having to use the intervention, rejection and discontinuance are likely [7]. The competence of individuals to judge the effectiveness of an intervention is facilitated by their understanding of underlying principles that justify using the intervention. When knowledge can be codified and transferred across contexts, implementation is more likely to be successful [1].

Enthusiastic use of an intervention requires a positive affective response to the intervention. Often, subjective opinions obtained from peers, based on personal experiences are more accessible and convincing and these opinions help to generate enthusiasm (more so than objective evidence requiring cognitive responses) [21]. The converse can be true as well, however, creating a negative source of active or passive resistance.

B. Self-efficacy – Individual belief in their own capabilities to execute courses of action to achieve implementation goals [80]. Self-efficacy is a significant component in most individual behavior change theories and models [68].

Self-efficacy is a key variable in most individual behavior change models [68]. Stakeholders with high self-efficacy are more likely to make a decision to embrace the intervention and exhibit committed use even in the face of obstacles. However, if they are not confident in their ability to use the intervention or experience a level of failure early-on, they will not be motivated to persist in the face of challenges that may arise [68].

C. Individual Stage of Change - Characterization of the phase an individual is in, as he or she progresses toward skilled, enthusiastic, and sustained use of the intervention [14].

Stage of change of individuals is an important measure of implementation progress and indicator of what kinds of engagement and educational strategies will be needed

for effective implementation. The CFIR is agnostic to which of the many models of stages of change are used to assess this construct. Some aspects of stages may overlap with knowledge and beliefs, described above. Grol et al outline 10 different taxonomies of stages and then propose their own 5-stage model, each with 2 sub-stages [14].

D. Individual Identification with Organization - A broad construct related to how individuals perceive the organization and their relationship and degree of commitment with that organization. These attributes may affect the willingness of staff to fully engage in implementation efforts or use the intervention [81-84].

This collection of measures has not been studied widely in healthcare. However, the organizational literature does include several concepts in studies of organizational change that may be interrelated or have independent influence on implementation. Because this is a relatively new constellation of masures, we included rationale for its inclusion in the main paper.

V. Process

The single most difficult domain to define, measure, or evaluate in implementation research is process. Many theories abound in just *how* implementation (or change) should be enacted embodied by theories of total quality management, integrated care, complexity theory, organizational learning, and others [14]. These approaches may be somewhat prescriptive, geared toward "what works." The CFIR's main goal is to foster knowledge-building into *why* implementations succeed or fail [85]. The CFIR is agnostic to any particular prescribed implementation approach. However, most approaches have four components in common (though sometimes only implicitly): planning, engaging, executing, and reflecting and evaluating. We provide relatively broad definitions for each of these areas because there are many process paths to effective implementation; from pre-planned sequential steps to rapid cycle improvements with quick and brief plans for small incremental tests to covert grass-roots efforts. The process of implementation is transient, by definition. Small efforts may produce deep and broad improvements while large efforts may result in little to no improvements [53]. We argue that regardless of where any particular implementation process lays on this continuum, success is most likely in the presence of these four constructs. The four constructs together echo the four component of the PDSA cycle that is a part of the quality improvement paradigm [86]: Plan, Do (execute), Study (reflect and evaluate), and Act (adjust the plan and/or execute, as appropriate). We have added engagement as a highlighted part of a cyclical approach because of the importance of engaging key individuals strategically in the process. The four constructs, plan, engage, execute, along with reflect and evaluate are often done in an incremental or spiral approach to implementation – there may be fits and starts with many iterations of complete and incomplete cycles to accomplish implementation (as depicted in Figure 1 in the main paper).

The CFIR takes a broader view of process than is implied by the PARiHS model's use of "facilitation." Kitson, et al assert that facilitation should be informed by the findings of the content and context analysis [76]. The CFIR defines process that includes facilitation as one potential mechanism in the process of connecting an intervention and setting with effective implementation.

As stated in the main paper, rarely, are the four process activities done in order and are not meant to be sequential. Nearly always, these four activities are done in a spiral or incremental approach to implementation; each activity will be revisited, expanded, refined, and re-evaluated throughout implementation. These constructs can be studied from the perspective of an observer or can be used to guide planning and execution. The more effectively each of the four mechanisms is carried out, the more effective implementation will be.

A. Planning – The degree to which a scheme or method of behavior and tasks for implementing an intervention are developed in advance and quality of those schemes or methods.

Planning is one of four fundamental activities in the PSDA (Plan-Do-Study-Act) cycle for implementing change in quality improvement efforts [87]. An assumption underlying implementation models is that they guide planning. This is not always explicitly stated, however. Greenhalgh et al, describe a continuum of conceptual and theoretical bases for spread of innovations in organizations from "let happen," unpredictable, emergent adoption to "make it happen," scientific, planned implementation [1]. More research is needed into the role of planning and the nature of quality of planning and its influence on implementation.

B. Engaging – Attracting and involving appropriate individuals in the implementation and use of the intervention through a combined strategy of social marketing, education, role modeling, training, and other similar activities.

Engaging members of teams tasked with implementing an intervention (or to be "first users") is an often overlooked part of implementation. It is vital that early members are carefully and thoughtfully selected [24]. The positive influence of having the "right people in the right seats" [88] is strong. Likewise, having the wrong people or missing key opportunities to "engage" important individuals can have tremendous negative influence on implementation success. An approach used in some implementations, is simply to select people by virtue of their role in the organization or to select somewhat randomly. This approach may be effective in some situations but in others it squanders a valuable opportunity to build a cohesive team consisting of effective champions and stakeholders who are most likely to make the implementation a success. If supporters of the intervention outnumber and are better strategically positioned than opponents are, the implementation is more likely to be successful [1]. Involving all stakeholders (e.g., leadership, agents, users) early in implementation enhances success [1]. Engaging staff in meaningful problem-solving is one of five interactive elements critical to transform patient care [56].

Implementation will be more effective when key individuals are dedicated (in terms of time and energy), empowered, and supported in their efforts [15, 89, 90] and if they are homophilous (similar socioeconomic, professional, educational, and cultural backgrounds) with intended users [1]. Key individuals are those who rise up in any capacity to help shepherd the implementation process to completion. Implementation leaders may be opinion leaders, formally appointed internal implementation leaders, champions, and/or external change agents. The quality of support provided by these implementation leaders is positively associated with implementation [91]. Effectively identifying and engaging these key individuals early in the process is important [92]. These leadership roles are used inconsistently and sometimes synonymously in the literature [1]. It is important to provide clarity in

defining these roles. Implementation leaders may emerge somewhat organically through the course of implementation. For example, a leader may emerge out of a grass-roots (bottom-up) initiative to improve compliance with an infection control practice. Alternatively, implementation leaders may be identified through top-down appointment for example, by appointing local champions or a project coordinator.

The PARiHS model asserts that "the role of the appropriately prepared facilitator, along with the team(s) they are working with, is to construct a programme of change that meets individual and team learning needs" [76](p 22). The CFIR takes a broader view, recognizing the role of many different types of leaders and influencers. Specific roles and expectations will vary depending on the study and setting and even over time within a single implementation effort. We describe several roles that appear in the literature below. However, any one role cannot function in isolation; personal characteristics certainly matter but *relationships* between these individuals can be more important than individual roles or characteristics [93].

<u>B1. Opinion Leaders</u> – Individuals in an organization who have formal or informal influence on the attitudes and beliefs of their colleagues with respect to implementing the intervention [1, 21]. There is general agreement that there are 2 different types of opinion leaders: 1) experts; and 2) peers [94].

Rogers defines opinion leaders in terms of the degree to which an individual is able to influence other attitudes or overt behavior informally in a desired way [21]. But opinion leaders can also exert a strong negative effect as well [94]. External change agents often use opinion leaders in a social system as "lieutenants" in diffusion activities [21]. Opinion leaders can lose respect of their peers if they come to be regarded as a professional change agent [21]. The role and definition of opinion leaders is varied and complex. Its effect on promoting use of interventions is mixed based on a review of randomized control trials ranging from -6% to +25% in improving behaviors of healthcare professionals [95].

<u>B2. Formally Appointed Internal Implementation leaders</u> – Individuals from within the organization who have been formally appointed with responsibility for implementing an intervention as coordinator, project manager, team leader, or other similar role.

This role includes individuals from within the organization who have been formally appointed with responsibility for implementation. These leaders may or many not have explicit time dedicated to the task but they will be more effective if they have dedicated time rather than as a distraction on top of other job duties [15]. For these leaders, implementation is "part of the job." Internal implementation leaders may also act as a champion or opinion leader, or may simply execute a plan or organize meetings between key individuals. Whether or not these leaders play a dual-role (e.g., a project coordinator who is also a champion) depends on the degree of passion, creativity, and willingness to take risks.

<u>B3. Champions</u> – "Individuals who dedicate themselves to supporting, marketing, and 'driving through' an [implementation]" [96](p. 182), overcoming indifference or resistance that the intervention may provoke in an organization.

The main distinction of champion from opinion leader is that a champion is *actively* involved with the implementation. There is adage that an intervention "either finds a champion or dies" [96]. A defining characteristic of champions is their willingness to

risk informal status and reputation because they believe so strongly in the intervention. Champions may occur at different levels of the organization: 1) Front-line champions who are most effective when they are able to defend and develop cross-functional coalitions within the organization of individuals who strongly believe in the intervention and are able to articulate the benefits in a way to move other individuals to fully embrace the intervention; and/or 2) supervisor or manager champions who empower front-line champion(s) and provide autonomy from rules, procedures and systems of the organization so the front-line champions can establish creative solutions to existing problems *and* who harnesses support from other members of the organization. Effective champions garner support from those in authority and/or build a broad base of support. There is strong to moderate support for the role of champions [1, 8, 21] though the literature is mixed on the influence of champions on implementation.

B4. External Change Agents – Individuals who are affiliated with an outside entity who formally influence or facilitate intervention decisions in a desirable direction.

External change agents usually have professional training in a technical field related to organizational change science or in the technology being introduced into the organization. This role includes outside researchers who may be implementing a multi-site intervention study and also includes other formally appointed individuals from an external entity (related or unrelated to the organization); e.g., a facilitator from the corporate or regional office.

The more external change agents actually do on behalf of the receiving organization, the more likely implementation will be successful in the near-term but the less likely the intervention can be sustained; the less they do, the less effective implementation is likely to be in the near-term but more effective over the longer term because local individuals may take more ownership. The PARiHS framework describes facilitators who are task-oriented versus holistic, the latter approach being valued more highly than the former [5].

C. Executing – Carrying out or accomplishing the implementation according to plan

Clearly execution of implementation plans and tasks is necessary for effective implementation. Quality of execution may consist of the fidelity of implementation to planned courses of action, timeliness of task completion, and degree of engagement of key involved individuals (e.g., implementation leaders) in the implementation process. There is not a consensus in the literature about how to assess fidelity – especially how to do so in a standardized way across studies and settings; it may not be appropriate to do so. Pearson, et al defined fidelity in terms of the extent to which hard core components of the intervention were implemented and also, quality and depth of implementation (together comprising intensity of implementation) [97]. The latter dimension (intensity) was subjectively determined based on organizational documents, meetings, communications, and other study materials.

Execution of an implementation plan can be organic with no obvious or formal planning which makes this construct challenging to assess. However, in addition to a well developed plan, three general approaches increase the likelihood of success. Dry runs are simulations or practice sessions that allow team members to learn how to use the intervention before going live and help train and prepare users [24]. Pilots or trials of a new intervention also allow users to test procedures, gain confidence, and build an environment of psychological safety as members are encouraged to take on

new behaviors, and make adjustments [24]. More complex interventions may lead to incremental interventions [35, 98, 99]. Breaking down the intervention into manageable parts that can be adopted incrementally has a positive relationship with adoption [35] because incremental implementation allows people to feel that they have enough time to do their work and to learn new skills associated with the shift to the new intervention [8]. Successes in early increments of the implementation help increase confidence, give an opportunity to make adjustments, and gain new "believers."

D. Reflecting and Evaluating - Quantitative and qualitative feedback about the progress and quality of implementation accompanied with regular personal and team debriefing about progress and experience.

Theoretical and Empirical Support
This is another often overlooked part of an effective implementation process. Evaluation includes traditional forms of feedback such as reports and graphs and also non-quantitative feedback including anecdotal stories of success. This kind of feedback is an essential component captured in many individual behavior change theories and models [68] and has strong to moderate evidence at an organizational level [1]. Less attention is paid, in the literature, to the need for group and personal reflection. Time must be taken to reflect or "debrief" before, during, and after implementation. These times of reflection help foster a learning climate – one in which a successful implementation can be ingrained into institutional memory and help improve the odds for future implementations [24] [34]. Even failures, when reflected upon in an effective way, can lead to future success when the root causes for the failure are uncovered in a psychologically safe and open way [44].

1. Greenhalgh T, Robert G, Macfarlane F, Bate P, Kyriakidou O: **Diffusion of innovations in service organizations: systematic review and recommendations.** *Milbank Q* 2004, **82:**581-629.
2. Rabin BA, Brownson RC, Haire-Joshu D, Kreuter MW, Weaver NL: **A glossary for dissemination and implementation research in health.** *J Public Health Manag Pract* 2008, **14:**117-123.
3. Van de Ven AH, Polley DE, Garud R, Vandataraman S: *The Innovation Journey.* Oxford: Oxford University Press; 1999.
4. Kitson A, Harvey G, McCormack B: **Enabling the implementation of evidence based practice: a conceptual framework.** *Qual Health Care* 1998, **7:**149-158.
5. Rycroft-Malone J, A., Kitson G, Harvey B, McCormack K, Seers AT, Estabrooks C: **Ingredients for change: revisiting a conceptual framework. (Viewpoint).** *Quality and Safety in Health Care* 2002, **11:**174-180.
6. Gustafson DH, Sainfort F, Eichler M, Adams L, Bisognano M, Steudel H: **Developing and testing a model to predict outcomes of organizational change.** *Health Serv Res* 2003, **38:**751-776.
7. Klein KJ, Conn AB, Sorra JS: **Implementing computerized technology: An organizational analysis.** *J Appl Psychol* 2001, **86:**811-824.
8. Helfrich CD, Weiner BJ, McKinney MM, Minasian L: **Determinants of implementation effectiveness: adapting a framework for complex innovations.** *Med Care Res Rev* 2007, **64:**279-303.
9. Dopson S, FitzGerald L, Ferlie E, Gabbay J, Locock L: **No magic target! Changing clinical practice to become more evidence based.** *Health Care Manage Rev* 2002, **27:**35-47.
10. Fitzgerald L, Dopson S: **Knowledge, credible evidence, and utilization.** In *Knowledge to action? Evidence-based health care in context.* Edited by Dopson S, Fitzgerald L. Oxford, UK: Oxford University Press; 2006: 223
11. Stetler CB: **Updating the Stetler Model of research utilization to facilitate evidence-based practice.** *Nurs Outlook* 2001, **49:**272-279.
12. Rycroft-Malone J, Harvey G, Kitson A, McCormack B, Seers K, Titchen A: **Getting evidence into practice: ingredients for change.** *Nurs Stand* 2002, **16:**38-43.
13. Graham ID, Logan J: **Innovations in knowledge transfer and continuity of care.** *Can J Nurs Res* 2004, **36:**89-103.
14. Grol RP, Bosch MC, Hulscher ME, Eccles MP, Wensing M: **Planning and studying improvement in patient care: the use of theoretical perspectives.** *Milbank Q* 2007, **85:**93-138.
15. Feldstein AC, Glasgow RE: **A practical, robust implementation and sustainability model (PRISM) for integrating research findings into practice.** *Jt Comm J Qual Patient Saf* 2008, **34:**228-243.
16. Denis JL, Hebert Y, Langley A, Lozeau D, Trottier LH: **Explaining diffusion patterns for complex health care innovations.** *Health Care Manage Rev* 2002, **27:**60-73.
17. Meyer AD, Goes JB: **Organisational Assimilation of Innovations: A Multi-Level Contextual Analysis.** *Acad Manage J* 1988, **31:**897-923.
18. Tucker AL, Nembhard I, Edmondson. AC: **Implementing New Practices: An Empirical Study of Organizational Learning in Hospital Intensive Care Units.** *Management Science* 2007, **53:**894-907.
19. Berta WB, Baker GR: **Factors that Impact the Transfer and Retention of Best Practices for Reducing Error in Hospitals.** *Health Care Manage Rev* 2004, **29:**90-97.
20. Perrin KM, Burke SG, O'Connor D, Walby G, Shippey C, Pitt S, McDermott RJ, Forthofer MS: **Factors contributing to intervention fidelity in a multi-site chronic disease self-management program.** *Implement Sci* 2006, **1:**26.
21. Rogers E: *Diffusion of Innovations.* 5 edn. New York, NY: Free Press; 2003.
22. Leeman J, Baernholdt M, Sandelowski M: **Developing a theory-based taxonomy of methods for implementing change in practice.** *J Adv Nurs* 2007, **58:**191-200.

23. Kochevar LK, Yano EM: **Understanding health care organization needs and context. Beyond performance gaps.** *J Gen Intern Med* 2006, **21 Suppl 2:**S25-29.
24. Edmondson AC, Bohmer RM, Pisana GP: **Disrupted routines: Team learning and new technology implementation in hospitals.** *Adm Sci Q* 2001, **46:**685-716.
25. Teplensky JD, Pauly MV, Kimberly JRH, Alan L., Schwartz JS: **Hospital adoption of medical technology: an empirical test of alternative models.** *Health Serv Res* 1995, **30:**437(429).
26. Institute of Medicine: *Crossing the Quality Chasm: A New Health System for the 21st Century.* Washington, DC: National Academy Press; 2001.
27. Ferlie EB, Shortell SM: **Improving the quality of health care in the United Kingdom and the United States: a framework for change.** *Milbank Q* 2001, **79:**281-315.
28. Squires JE, Moralejo D, Lefort SM: **Exploring the role of organizational policies and procedures in promoting research utilization in registered nurses.** *Implement Sci* 2007, **2:**17.
29. Shortell SM, Marsteller JA, Lin M, Pearson ML, Wu SY, Mendel P, Cretin S, Rosen M: **The role of perceived team effectiveness in improving chronic illness care.** *Med Care* 2004, **42:**1040-1048.
30. Barnsley J, Lemieux-Charles L, McKinney MM: **Integrating learning into integrated delivery systems.** *Health Care Manage Rev* 1998, **23:**18-28.
31. Kimberly JR, Evanisko MJ: **Organizational innovation: the influence of individual, organizational, and contextual factors on hospital adoption of technological and administrative innovations.** *Acad Manage J* 1981, **24:**689-713.
32. Aiken M, S.B. Bacharach and J.L. French. : **Organizational Structure, Work Process, and Proposal making in Administrative Bureaucracies.** *The Academy of Management Journal* 1980, **23:**631-652.
33. Baldridge JV, Burnham RA: **Organizational Innovation: Individual, organizational and environment impacts.** *Adm Sci Q* 1975, **20:**165-176.
34. Simpson DD, Dansereau DF: **Assessing Organizational Functioning as a Step Toward Innovation.** *NIDA Science & Practice Perspectives* 2007, **3:**20-28.
35. Damanpour F: **Organizational Innovation: A Meta-Analysis of Effects of Determinants and Moderators.** *The Academy of Management Journal* 1991, **34:**555-590.
36. Burns LR, Wholey DR: **Adoption and Abandonment of Matrix Management Programs: Effects of Organizational Characteristics and Interorganizational Networks.** *Acad Manage J* 1993, **36:**106-138.
37. Gladwell M: *The Tipping Point.* New York: Little, Brown and Company; 2000.
38. Brehem J, Rahn W: **Individual Level Evidence for the Causes and Consequences of Social Capital.** *American Journal of Political Science* 1997, **41:**999-1023.
39. Gittell R, Vidal V: *Community Organizing: Building Social as a Development Strategy.* Thousand Oaks, CA: Sage Publications; 1998.
40. Leana CR, Pil FK: **Social Capital and Organizational Performance: Evidence from Public Schools.** *Organization Science* In Press, **17:**353-366.
41. Frambach RT, Schillewaert N: **Organizational innovation adoption: a multi-level framework of determinants and opportunities for future research.** *Journal of Business Research* 2001, **55:**163-176.
42. Walston SL, Kimberly JR, Burns LR: **Institutional and economic influences on the adoption and extensiveness of managerial innovation in hospitals: The case of reengineering.** *Med Care Res Rev* 2001, **58:**194-228; discussion 229-133.
43. Mendel P, Meredith LS, Schoenbaum M, Sherbourne CD, Wells KB: **Interventions in organizational and community context: a framework for building evidence on dissemination and implementation in health services research.** *Adm Policy Ment Health* 2008, **35:**21-37.
44. Klein KJ, Sorra JS: **The Challenge of Innovation Implementation.** *The Academy of Management Review* 1996, **21:**1055-1080.

45. Dopson S, Fitzgerald L: **The active role of context.** In *Knowledge to action: Evidence-based health care in context.* Edited by Dopson S, Fitzgerald L. Oxford: Oxford University Press; 2005: 79-103
46. Thompson J, Scott W, Zald M: *Organizations in Action: Social Science Bases of Administrative Theory.* Edison, NJ: Transaction Publishers; 2003.
47. Pugh DS, Hickson DJ, Hinings CR, C.Turner: **Dimensions of Organization Structure.** *Adm Sci Q* 1968, **13:**65-105.
48. Dewar RD, Dutton JE: **The Adoption of Radical and Incremental Innovations: An Empirical Analysis.** *Management Science* 1986, **32:**1422-1433.
49. Hull FM, Hage J: **Organizing for innovation: Beyond Burns and Stalker's organic type.** *Sociology* 1982, **16:**564-577.
50. Van de Ven AH: **Central Problems in the Management of Innovation.** *Management Science* 1986, **32:**590-607.
51. Scott J: *Social Network Analysis: A Handbook.* 2nd edn. London, UK: Sage Publications; 2000.
52. Fitzgerald LA, van Eijnatten FM: **Reflections: Chaos in organizational change.** *Journal of Organizational Change Management* 2002, **15:**402-411.
53. Burnes B: **Complexity theories and organizational change.** *International Journal of Management Reviews* 2005, **7:**73-90.
54. Lincoln YS, Guba EG: **Paradigmatic controversies, contradictions, and emerging confluences.** In *The Landscape of Qualitative Research: Theories and Issues.* 2nd edition. Edited by Denzin NK, Lincoln YS. Thousand Oaks, CA: Sage Publications; 2003: 253-291
55. Fitzgerald LA: **Chaos: the lens that transcends.** *Journal of Organizational Change Management* 2002, **15:**339-358.
56. VanDeusen Lukas CV, Holmes SK, Cohen AB, Restuccia J, Cramer IE, Shwartz M, Charns MP: **Transformational change in health care systems: An organizational model.** *Health Care Manage Rev* 2007, **32:**309-320.
57. Bodenheimer T, Wagner EH, Grumbach K: **Improving primary care for patients with chronic illness: the chronic care model, Part 2.** *JAMA* 2002, **288:**1909-1914.
58. Bodenheimer T, Wagner EH, Grumbach K: **Improving primary care for patients with chronic illness.** *JAMA* 2002, **288:**1775-1779.
59. Pronovost PJ, Berenholtz SM, Goeschel CA, Needham DM, Sexton JB, Thompson DA, Lubomski LH, Marsteller JA, Makary MA, Hunt E: **Creating high reliability in health care organizations.** *Health Serv Res* 2006, **41:**1599-1617.
60. Meyers PW, Sivakumar K, Nakata C: **Implementation of Industrial Process Innovations: Factors, Effects, and Marketing Implications.** *Journal of Product Innovation Management* 1999, **16:**295-311.
61. Gershon R, Stone PW, Bakken S, Larson E: **Measurement of Organizational Culture and Climate in Healthcare.** *J Nurs Adm* 2004, **34:**33-40.
62. Shortell SM, Zazzali JL, Burns LR, Alexander JA, Gillies RR, Budetti PP, Waters TM, Zuckerman HS: **Implementing evidence-based medicine: the role of market pressures, compensation incentives, and culture in physician organizations.** *Med Care* 2001, **39:**I62-78.
63. Helfrich C, Li Y, Mohr D, Meterko M, Sales A: **Assessing an organizational culture instrument based on the Competing Values Framework: Exploratory and confirmatory factor analyses.** *Implementation Science* 2007, **2:**13 [ePub, ahead of print].
64. Eijnatten FM, Galen M: **Chaos, dialogue and the dolphin's strategy.** *Journal of Organizational Change Management* 2002, **15:**391-401.
65. Quinn R, Rohrbaugh J: **A Competing Values Approach to Organizational Effectiveness.** *Public Productivity Review :* 1981**:**122-140.
66. Lehman WE, Greener JM, Simpson DD: **Assessing organizational readiness for change.** *J Subst Abuse Treat* 2002, **22:**197-209.
67. Auber BA, Hamel G: **Adoption of smart cards in the medical sector: the Canadian experience.** *Soc Sci Med* 2001, **53:**879-894.

68. **Theory at a Glance: A guide for health promotion practice** [http://www.cancer.gov/PDF/481f5d53-63df-41bc-bfaf-5aa48ee1da4d/TAAG3.pdf]
69. Jamtvedt G, Young JM, Kristoffersen DT, O'Brien MA, Oxman AD: **Does telling people what they have been doing change what they do? A systematic review of the effects of audit and feedback.** *Qual Saf Health Care* 2006, **15:**433-436.
70. Hysong SJ, Best RG, Pugh JA: **Audit and feedback and clinical practice guideline adherence: Making feedback actionable.** *Implement Sci* 2006, **1:**9.
71. Nembhard I, Edmonson A: **. Making it safe: the effects of leader inclusiveness and professional status on psychological safety and improvement efforts in health care teams.** *Journal of Organizational Behavior* 2006, **27:**941-966.
72. Edmondson AC: **The local and variegated nature of learning in organizations: A group-level perspective.** *Organization Science* 2002, **13** 128-146.
73. Goh S, Richards G: **Benchmarking the learning capability of organizations.** *European Management Journal* 1997, **15:**575-583.
74. Templeton GF, Lewis BR, Snyder CA: **Development of a measure for the organizational learning construct.** *Journal of Management Information Systems* 2002, **19:**175-218.
75. Simpson DD: **A conceptual framework for transferring research to practice.** *J Subst Abuse Treat* 2002, **22:**171-182.
76. Kitson AL, Rycroft-Malone J, Harvey G, McCormack B, Seers K, Titchen A: **Evaluating the successful implementation of evidence into practice using the PARIHS framework: theoretical and practical challenges.** *Implement Sci* 2008, **3:**1.
77. Repenning NP: **A simulation-based approach to understanding the dynamics of innovation implementation.** *Organization Science* 2002, **13:**109-127.
78. Singh J, Lumsden CJ: **Theory and research in organizational ecology.** *Annual Review of Sociology* 1990, **16:**161-195.
79. Wallin L, Estabrooks CA, Midodzi WK, Cummings GG: **Development and validation of a derived measure of research utilization by nurses.** *Nurs Res* 2006, **55:**149-160.
80. Bandura A: **Self-efficacy: toward a unifying theory of behavioral change.** *Psychol Rev* 1977, **84:**191-215.
81. Greenberg J: **Organizational justice: Yesterday, today, and tomorrow.** *Journal of Management* 1990, **16:**399-432.
82. Abraham R: **Organizational cynicism: bases and consequences.** *Genet Soc Gen Psychol Monogr* 2000, **126:**269-292.
83. Cummings GG, Estabrooks CA, Midodzi WK, Wallin L, Hayduk L: **Influence of organizational characteristics and context on research utilization.** *Nurs Res* 2007, **56:**S24-39.
84. Estabrooks CA, Midodzi WK, Cummings GG, Wallin L: **Predicting research use in nursing organizations: a multilevel analysis.** *Nurs Res* 2007, **56:**S7-23.
85. Institute of Medicine (IOM): *The state of quality improvement and implementation research: Expert views. Workshop summary.* Washington, DC: The National Academies Press; 2007.
86. IHI: **The Breakthrough Series: IHI's Collaborative Model for Achieving Breakthrough Improvement.** In *Innvation Series 2003*. pp. 20. Cambridge, MA: Institutue for Healthcare Improvement; 2003:20.
87. Institute for Healthcare Improvement: **The Breakthrough Series: IHI's Collaborative Model for Achieving Breakthrough Improvement.** In *Innvation Series 2003*. pp. 20. Cambridge, MA: Institutue for Healthcare Improvement; 2003:20.
88. Collins J: *Good to Great: Why Some Companies Make the Leap... and Others Don't* New York, NY: HarperBusiness; 2001.
89. Brach C, Lenfestey N, Roussel A, Amoozegar J, Sorensen A: *Will It Work Here? A Decisionmaker's Guide to Adopting Innovations.* Agency for Healthcare Research & Quality (AHRQ); 2008.
90. Fixsen DL, Naoom, S. F., Blase, K. A., Friedman, R. M. & Wallace, F.: **Implementation Research: A Synthesis of the Literature.** (The National Implementation Research

Network ed.: University of South Florida, Louis de la Parte Florida Mental Health Institute; 2005.

91. Ovretveit J, Bate P, Cleary P, Cretin S, Gustafson D, McInnes K, McLeod H, Molfenter T, Plsek P, Robert G, et al: **Quality collaboratives: lessons from research.** *Qual Saf Health Care* 2002, **11:**345-351.

92. Pronovost PJ, Berenholtz SM, Needham DM: **Translating evidence into practice: a model for large scale knowledge translation.** 2008, **337:**a1714-.

93. Plsek PE, Wilson T: **Complexity, leadership, and management in healthcare organisations.** *BMJ* 2001, **323:**746-749.

94. Locock L, Dopson S, Chambers D, Gabbay J: **Understanding the role of opinion leaders in improving clinical effectiveness.** *Soc Sci Med* 2001, **53:**745-757.

95. Doumit G, Gattellari M, Grimshaw J, O'Brien MA: **Local opinion leaders: effects on professional practice and health care outcomes.** *Cochrane Database Syst Rev* 2007**:**CD000125.

96. Greenhalgh T, Robert G, Bate P, Kyriakidou O, Macfarlane F, Peacock R: **How to Spread Good Ideas.** (NCCSDO ed. pp. 424. London, England: National Co-ordinating Centre for NHS Service Delivery and Organisation R & D; 2004:424.

97. Pearson ML, Wu S, Schaefer J, Bonomi AE, Shortell SM, Mendel PJ, Marsteller JA, Louis TA, Rosen M, Keeler EB: **Assessing the implementation of the chronic care model in quality improvement collaboratives.** *Health Serv Res* 2005, **40:**978-996.

98. Grossman JB: **The Supreme Court and Social Change: A Preliminary Inquiry.** *Am Behav Sci* 1970, **13:**535-551.

99. Normann R: **Organizational Innovativeness: Product variation and reorientation.** *Adm Sci Q* 1971, **16**.
